# Comparative metagenomic assessment of Illumina-compatible library preparation methods, short-read lengths, and PacBio HiFi sequencing reveals differences in microbial and functional diversity recovery from a complex environmental sample

**DOI:** 10.1128/spectrum.00013-26

**Published:** 2026-07-27

**Authors:** Rubén Díaz-Rúa, Daniela I. Drautz-Moses, Xiang Zhao, Sadhasivam Perumal, Luke Esau, Angel Angelov, Alexander Putra, Patrick Driguez, Ming Sin Cheung, Emanuele Palescandolo

**Affiliations:** 1 Bioscience Core Labs (BCL), King Abdullah University of Science and Technology (KAUST) 127355, Thuwal, Saudi Arabia; The Ohio State University, Columbus, Ohio, USA

**Keywords:** microbiome, metagenomics, environmental microbiology

## Abstract

Metagenomics enables comprehensive exploration of microbial communities but is influenced by library preparation and sequencing technologies, affecting recovery of microbial genomes and proteins. Here, we benchmarked six Illumina-compatible short-read library preparation conditions in triplicate at 2 × 150 bp and 2 × 250 bp read lengths alongside PacBio HiFi long-read sequencing using a composite environmental sample of marine mangrove sediment and terrestrial palm tree soil. Longer short reads (2 × 250 bp) combined with optimal library preparation approaches improved assembly quality, protein detection, and metagenome-assembled genome (MAG) recovery, achieving results approaching those of long-read sequencing. TruSeq libraries at 2 × 250 bp recovered more than sevenfold more unique proteins than the same kit at 2 × 150 bp (811,701 vs 110,108) using the same number of sequencing reads, while recovering a comparable number of high-quality MAGs to PacBio HiFi long-read sequencing (11 vs 18) and surpassing it in protein discovery by almost 10-fold (811,701 vs 87,745) at less than half of the sequencing cost. Furthermore, biosynthetic gene cluster analysis identified 46 biosynthetic gene clusters in TruSeq-250PE assemblies compared to 38 in PacBio HiFi, with several showing no close match in the MIBiG database. Although long reads yield more contiguity and complete genomes, longer short reads offer a cost-effective, scalable alternative for uncovering microbial and functional diversity. These findings provide critical guidance for metagenomic experimental design, demonstrating that strategic selection of library preparation chemistry and sequencing parameters can reveal more unknown microbial information in complex biomes without requiring additional sequencing depth.

**IMPORTANCE:**

Metagenomic outcomes are strongly influenced by library preparation and sequencing strategies, yet their combined effects in complex environmental samples remain poorly defined. Here, we provide the first direct comparison of Illumina NovaSeq short-read metagenomic sequencing at 2 × 150 bp and 2 × 250 bp across multiple library preparation kits, alongside PacBio HiFi long-read sequencing. We show that sequencing read length and library preparation critically shape assembly quality, protein recovery, and metagenome-assembled genome (MAG) reconstruction. These findings demonstrate that short-read sequencing at 2 × 250 bp, with appropriate library preparation, can match long-read technologies in MAG recovery while substantially surpassing them in protein discovery. With less than half of the sequencing price and a 3.5-fold reduction in cost per gigabase of usable data, this method facilitates more accessible large-scale metagenomic analysis within complex environmental systems.

## INTRODUCTION

Sequencing technologies have changed the landscape of research in the life sciences, ushering in a new era of genomic discovery (1). These advancements have also significantly enhanced our capacity to address ecological and evolutionary questions, enabling comprehensive genetic surveys across the entire tree of life (2). The application of high-throughput sequencing for studying microbial communities has established metagenomics as a scientific approach for analyzing the collective genomes of organisms coexisting within a given environment. At its core, metagenomics explores genetic material recovered directly from environmental samples, providing a holistic view of entire microbial communities (3, 4). This approach bypasses the need to isolate and cultivate individual microorganisms, including the unknown and uncultivated fraction, termed the “dark matter” (5).

Harvesting microbial information from metagenomes has significantly contributed to the discovery of novel enzymes (6), drug candidates from marine products (7, 8), and industrial applications (9). However, the outcomes of metagenomic studies are often influenced by variations in experimental protocols. Factors such as DNA extraction methods (10, 11), library preparation approaches (12), sequencing platforms (13), sequencing depth, and read length can significantly influence the quantitative and qualitative characterization of microbial communities. This underscores the need for standardized and robust protocols to ensure comparability across studies, with library construction method playing a critical role (14). While previous studies testing library preparation methods have largely emphasized differences in sequencing (e.g., GC content and PCR duplicates) and taxonomic traits, identification of novel and unknown proteins and the characterization of non-cultivable microorganisms are often not adequately addressed.

Sequencing technologies are commonly classified as (i) short-read and high-throughput (<300 bp) or next-generation sequencing (NGS) and (ii) long-read (>300 bp), with the latter facing limitations in throughput, cost, and DNA quality and quantity requirements (15) but often leading to improved genome assembly (16). Although 2 × 150 bp reads are widely employed in metagenomics, and prior studies have examined the impact of read length through simulations and computational comparisons (17, 18), empirical, platform-specific assessments of metagenomics performance comparing different read lengths by sequencing the same library under otherwise identical conditions on the Illumina NovaSeq 6000 remain scarce.

Current benchmarks in marine (13) and host-associated environments (16, 19, 20) have significantly advanced our understanding of sequencing performance. However, these studies typically rely on a single library preparation kit and a fixed short-read length. While the Illumina application note (21) compares multiple kits, it lacks long-read benchmarking, statistical validation, and functional assessments such as metagenome-assembled genome (MAG) discovery or biosynthetic potential. Crucially, no study to date has evaluated the combined effects of kit chemistry, read length, and DNA input concentration within a single complex environmental sample.

Furthermore, variations in environmental sample composition and experimental protocols pose a significant challenge to reproducibility. In comparative studies, while commercial mock bacterial communities are useful for benchmarking purposes, their limited species diversity compared to natural microbiomes may not be sufficient to detect protocol differences (12). In addition, the matrix in which microbial communities are often embedded (e.g., soil, coral, or biofilm), and its associated inhibitors, such as humic acids, polysaccharides, and calcium ions, among others, can add additional challenges to successful sample processing and reproducibility (11, 12, 22, 23).

In this study, we conducted comparative benchmarking of metagenomic sequencing strategies to assess the impact of library preparation, read length, and sequencing technology on the recovery of microbial information from complex environmental communities. We systematically compared four short-read sequencing (SRS) library preparation kits encompassing three distinct chemistries, tested at one or two DNA input levels (1 and/or 100 ng) where the kit protocol permitted, and at two sequencing read lengths (2 × 150 bp and 2 × 250 bp). To benchmark against long-read sequencing (LRS), we processed the same DNA sample with PacBio HiFi, covering a total of 13 distinct sequencing conditions. This design enabled us to assess the impact of library construction, sequencing read length, and technology on taxonomic profiling, protein detection, and the recovery of high-quality metagenome-assembled genomes.

## MATERIALS AND METHODS

### Sample preparation and DNA extraction

A diverse composite sample of mangrove sediment and palm tree soil was assembled to provide sufficient DNA quantity and quality for comprehensive testing across various library preparation kits and sequencing technologies without being influenced by different sampling sites and DNA extraction methods. This pooling strategy was adopted to ensure sufficient microbial complexity for discriminatory benchmarking, given that samples of low diversity have been shown to mask protocol-dependent differences (24) and that arid environments such as our sampling sites are known to harbor reduced microbial diversity (25, 26).

Samples were collected on 25 June 2021 from two distinct environments located inside the King Abdullah University of Science and Technology (KAUST) campus (Thuwal, Saudi Arabia): marine mangrove sediments (22°19′19.7″N, 39°06′08.7″E) and terrestrial palm tree soil (22°18′26.7″N, 39°06′16.0″E). Samples were collected from the upper 2 cm of the soil/sediment surface using sterilized spatulas, placed into sterilized 50 mL conical tubes, and processed immediately after collection without prior drying. A manual mixing step was performed prior to weighing and DNA extraction. For each environment, five independent extractions were carried out with the DNeasy PowerMax Soil Kit (Qiagen, Hilden, Germany), following the manufacturer’s instructions and using 8 g of mixed material per extraction. The large amount of material used per replicate ensured a high representation of microbes from both sites. Subsequently, the DNA aliquots from all extractions were pooled equimolarly in a single aliquot, which was then cleaned and concentrated using the NucleoSpin gDNA Clean-up Kit (MACHEREY-NAGEL, Düren, Germany).

### Library preparation and sequencing

For short-read sequencing, the following library preparation kits were used: NEBNext Ultra II DNA Library Prep Kit for Illumina (NEB, Ipswich, MA, USA), Nextera XT DNA Library Preparation Kit (Illumina, San Diego, CA, USA), Ovation Ultralow System V2 (Tecan, Männedorf, Switzerland), and TruSeq DNA Nano (Illumina, San Diego, CA, USA). Libraries were prepared in triplicate following the manufacturers’ instructions, and when the protocol allowed, we tested different DNA inputs, 1 and 100 ng (see Table 1 for all library preparation details). Size distribution was assessed using the Bioanalyzer 2100 (Agilent Technologies, Santa Clara, CA, USA). The libraries were then quantified using the KAPA Library Quantification Kit for Illumina Platforms (Roche, Basel, Switzerland). After equimolar pooling of all libraries, 1 nM aliquots were sequenced on NovaSeq 6000 SP flow cells at read lengths of 2 × 150 bp and 2 × 250 bp (referred to hereinafter as 150PE and 250PE, respectively).

**TABLE 1:** Library preparation kits used in this study

Commercial kit name	Sample name (replicate)	Co-assembly name	Fragmentation method	Input (ng)	Library price ($)	Sequencing price ($)	Assay time (h)
NEBNext Ultra II DNA Library Prep Kit for Illumina	NEB-1_1	NEB-1	Enzymatic	1	$40	150PE: $276	6
	NEB-1_2					250PE: $316	
	NEB-1_3						
NEBNext Ultra II DNA Library Prep Kit for Illumina	NEB-100_1	NEB-100	Enzymatic	100	$40	150PE: $276	6
	NEB-100_2					250PE: $316	
	NEB-100_3						
Nextera XT DNA Library Preparation Kit	NEXT-1_1	NEXT-1	Transposome	1	$65	150PE: $276	5
	NEXT-1_2					250PE: $316	
	NEXT-1_3						
Ovation Ultralow System V2	Ovation-1_1	Ovation-1	Sonication	1	$54	150PE: $276	6
	Ovation-1_2					250PE: $316	
	Ovation-1_3						
Ovation Ultralow System V2	Ovation-100_1	Ovation-100	Sonication	100	$54	150PE: $276	6
	Ovation-100_2					250PE: $316	
	Ovation-100_3						
TruSeq DNA Nano	TruSeq-100_1	TruSeq-100	Sonication	100	$50	150PE: $276	7
	TruSeq-100_2					250PE: $316	
	TruSeq-100_3						
Metagenomics Shotgun Library Preparation Using SMRTbell Express Template Prep Kit 2.0	PacBio	PacBio	Shearing	2,000	$281	$2,121	10

For LRS, 0.45× Ampure PB Beads (PacBio, Menlo Park, CA, USA) were used to concentrate the gDNA. DNA concentration and size distribution were determined using the Qubit HS dsDNA Assay Kit with the Qubit 2.0 Fluorometer (Life Technologies, CA, USA) and the Femto Pulse platform (Agilent Technologies, Santa Clara, CA, USA), respectively. The library was prepared with 2,000 ng of input DNA using the SMRTbell Express Template Prep Kit 2.0 (PacBio, Menlo Park, CA, USA). Final library concentration was measured using the Qubit dsDNA High Sensitivity Kit, and library size distribution was assessed with the Agilent Femto Pulse. Next, 100 pM of the SMRTbell library was mixed with the sequencing primer and Sequel II DNA polymerase from the Sequel II Binding kit 2.2 (101-894-200), following the SMRTlink sample set-up guidelines. HiFi sequencing was then performed with a 30-h movie time in adaptive loading mode, using the Sequel II Sequencing Kit 2.0 (101-820-200) and SMRT Cell 8M Tray (101-389-001).

All reagents and sequencing costs reported in this study reflect prices at the KAUST Bioscience Core Lab (BCL) at the time the experiments were performed and may vary across institutions and over time.

### Sequencing data analysis

**Fig 1:**
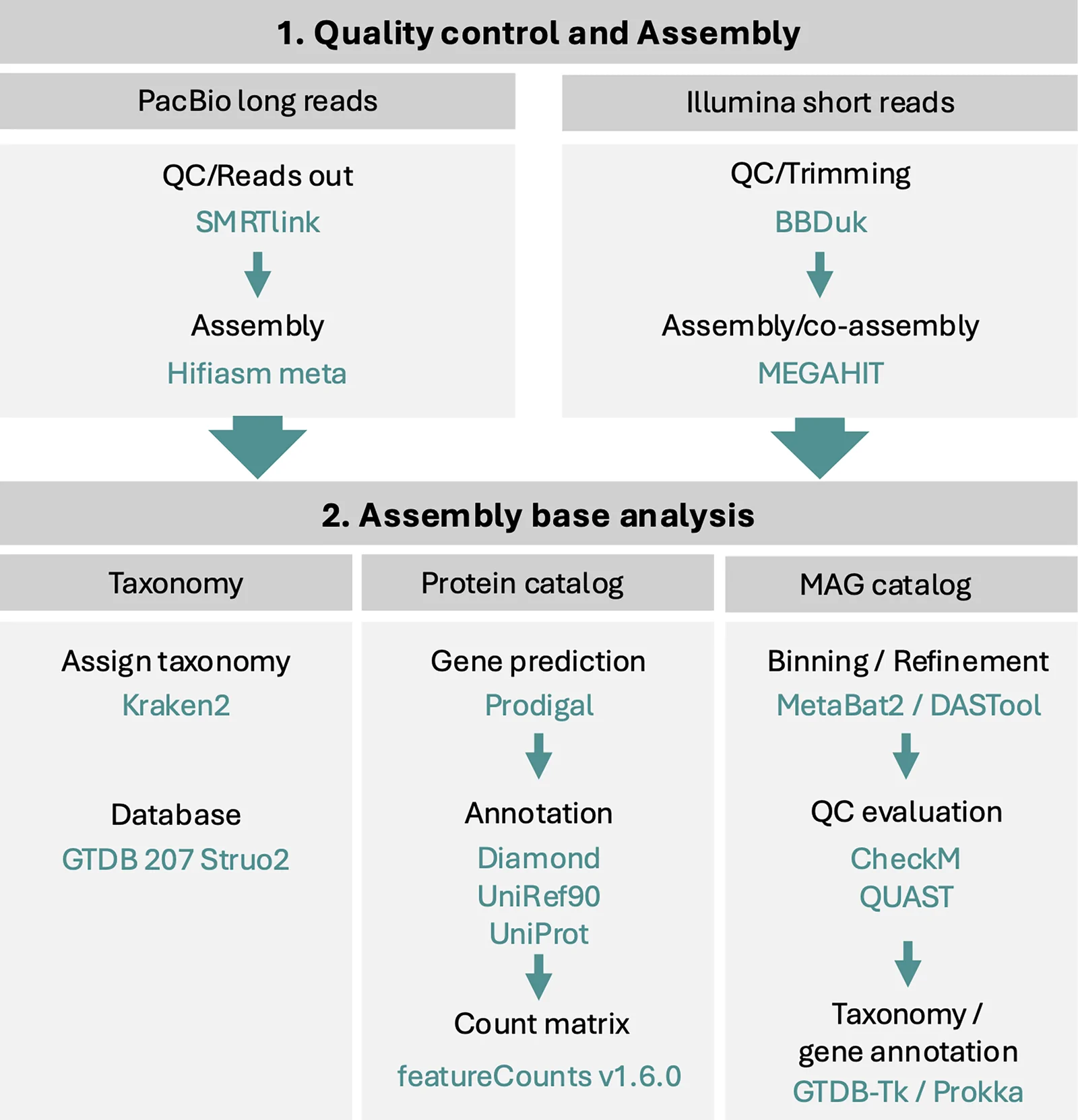
Overview of the sequencing data analysis pipeline used for both sequencing technologies.

Sequencing data were processed using standard metagenomic pipelines with specific tools and packages for quality control, adapter trimming, genome assembly, gene prediction, protein annotation, binning, and MAG characterization. Figure 1 provides an overview of the short- and long-read analysis pipelines.

#### Illumina short-read quality check, trimming, and assembly

BBDuk v39.08 from the BBTools package was used to trim adapters and remove low-quality sequences. Random downsampling was performed to ensure an equal number of reads across samples was used for subsequent analyses. Downsampled data were pooled per replicate, referred to as “kit merge” (KM), or pooled across all SRS kits and termed “global merge” (GM). High-quality reads were assembled using MEGAHIT v1.2.9 (27) with the option “--presets meta-large.” Assembly statistics, such as number of contigs, contig lengths, and N50/N90 metrics, were calculated using assembly-stats (GitHub repository sanger-pathogens/assembly-stats). Reads were mapped to the contigs using BWA v0.7.17-r1188 (28).

#### PacBio long-read quality check, trimming, and assembly

PacBio HiFi reads were quality-checked and filtered using the SMRTlink software v11 (PacBio, Menlo Park, CA, USA). Assembly was performed using the hifiasm meta-assembler (v0.3) (29) through the official PacBio pipeline (GitHub repository PacificBiosciences/pb-metagenomics-tools).

#### Taxonomic assignment and diversity analyses

Taxonomic classification for Illumina and PacBio contigs was performed using the Kraken2 v1.1.1 algorithm (30) within OmicsBox v3.4 (BioBam Bioinformatics, 2019) and the GTDB 207 Struo2 database (31). Beta diversity was assessed through principal coordinate analysis (PCoA) using Bray-Curtis dissimilarity matrix on the OTU matrix at the genus level for the SRS replicates. Significance of the resulting ordination was tested with permutational MANOVA (PERMANOVA) and 999 permutations (adonis2, vegan package v2.3-5 in R). Genus-level richness was calculated using the estimate_richness function from the phyloseq package v1.54.0 (32) in R 4.4.0 (33).

#### Differential abundance analysis of taxa

Differential abundance analysis at genus level was carried out with the edgeR package (34) in OmicsBox v3.4, applying a binomial generalized linear model with TMMwsp normalization using a minimum of 10 counts per million (CPM) and a minimum of 5 counts per sample to exclude low-abundance taxa that could overestimate the differences. Statistical differences between the groups were assessed using the Student’s *t*-test with multiple testing correction (false discovery rate, FDR) as previously described (35). Corrected *P*-values of <0.05 and with a log-fold change of ±1 were considered statistically significant. Significant genera were visualized in a heatmap where CPM values were presented on a logarithmic scale as *Z*-scores (36).

#### Gene prediction, annotation, and protein catalog

For both SRS and LRS data sets, gene prediction was conducted with Prodigal v2.6.3 (37) with the option “-p meta,” followed by a dereplication step of clustering at 95% amino acid identity with CD-HIT v4.8.1 (38) to remove genetic redundancy.

Subsequently, protein sequences obtained by CD-HIT were annotated against the UniRef90 database using Diamond blastp v2.1.8 (39), requiring a minimum read coverage of 90% and a minimum identity of 80%.

In order to decrease fragmented proteins that could inflate the results in large data sets such as the KM and GM co-assemblies, a more stringent filtering process was performed. Only contigs larger than 500 bp and genes longer than 100 bp (33 amino acids) were selectively retrieved. A unique set of proteins was identified using featureCounts v1.6.0 (40) to count the number of reads that mapped to each predicted protein. Proteins with a hit against the UniRef90 database (identity 80%) were then extracted, aggregating counts for proteins with the same UniProt IDs and keeping only proteins with more than one read. Protein hit information was downloaded from UniProt using UPIMAPI v1.13.0 (41). To assess the number of unique annotated and uncharacterized proteins and focus specifically on those with identified functions, a non-redundant protein catalog was generated by collapsing protein counts across the KM and GM data sets based on shared protein identifiers. Given its substantially higher sequencing depth—approximately six times greater than that of the KM co-assemblies—and its inclusion of reads from all Illumina-compatible library preparation conditions, the GM data set was selected as the reference protein database for this study.

#### Principal coordinate analysis for identified proteins in the Illumina kits

PCoA was performed on normalized gene count matrices (Bray-Curtis distances) using featureCounts v1.6.0 (40), with the GTF and BAM files for the individual samples obtained with Prodigal. The individual BAM files were generated by mapping the raw reads against the GM co-assembly. The count matrix was then normalized using the variance-stabilizing transformation. Genes with 0 variance were filtered out. Principal components were generated using the prcomp function in R on the Bray-Curtis distance. Statistical significance was tested with PERMANOVA.

#### MAG reconstruction, classification, and comparison

For MAG reconstruction, a binning process was carried out for the KM and GM co-assemblies and the PacBio data set. MetaBAT2 v2.15 (42) was used for this purpose, with a minimum contig length of 1,500 bp. After the binning, a refinement step was applied with DAS Tool v1.1.3 (43). MetaBAT2 was selected for binning across all sequencing conditions as it was, at the time of analysis, the most widely used tool compatible with both short- and long-read data, enabling consistent cross-technology comparisons, and has been recognized as one of the most computationally efficient binners across multiple data types and binning modes (42, 44). To validate that the observed differences were not influenced by the choice of binning algorithm, MetaWRAP v1.3 (which integrates MetaBAT2, MaxBin2, and CONCOCT) was additionally applied to all short-read KM data sets, with results presented in Table S1c (45). MetaWRAP was not applied to the PacBio long-read data set as it does not support long-read data. Completeness and contamination were performed with CheckM v1.2.2 (46). For benchmarking comparison, high-quality MAGs were defined as bins with completeness > 90% and contamination < 5%. PacBio bins were used as internal reference genomes, further serving as a benchmark for various short-read sequencing strategies. Taxonomic classification of MAGs with a completeness > 90% and a contamination < 5% was obtained with the Genome Taxonomy Database Toolkit (GTDB-Tk v1.5.0) (47). For high-quality PacBio MAGs with average nucleotide identities (ANIs) < 95% (standard speciation threshold) (48), a phylogenetic tree was constructed with the GTDB-Tk *de novo* workflow (de_novo_wf) in order to detect potential new microbes. The outgroup taxon (--outgroup_taxon) option was used to reduce the tree size (Fig. S3). In addition, the genome sequences were submitted to the Type (Strain) Genome Server (TYGS; https://tygs.dsmz.de) for whole-genome-based species delineation (49–51), using the digital DNA-DNA hybridization (dDDH) formula d4 and species and subspecies delineation thresholds of 70% and 79%, respectively.

For MAG comparison between sequencing technologies, the ANI between the SRS and LRS bins was determined using FastANI v1.32 (48), and the MASH distance and *P*-value were calculated to assess MAG similarity (52). QUAST v5.0 was used to determine assembly metrics (53), and Prokka v1.14.6 for gene annotation (54). To further consolidate the high quality of these selected MAGs, rRNA and tRNA gene annotation of the selected high-quality MAGs was performed using barrnap (v1.10.5; https://github.com/tseemann/barrnap). The results are summarized in Table S1d. In line with MIMAG standards (55), high-quality MAGs were additionally required to contain rRNA genes and a minimum of 18 tRNA genes. Visualization of each *de novo* MAG assembly was conducted with Bandage v0.8.1 (56). Orthologous gene clusters among the selected bins were identified using OrthoVenn3 (57) with default parameters. Finally, to explore the potential novel function of these high-quality MAGs, biosynthetic gene clusters were identified using antiSMASH version 8.0 (58) with default parameters. The tool was run in “full mode” to detect known and putative secondary metabolite clusters, including those for polyketide synthase (PKSs), nonribosomal peptide synthetase (NRPSs), terpenes, and others. Cluster boundaries, annotations, and predicted products were generated based on comparison with the MIBiG reference database (59).

### Statistical analysis and graphing

General statistical analysis and bar plots were performed using GraphPad Prism 9.0 for Mac. ANOVA followed by Tukey’s test was used to compare SRS library preparation kits. Student’s *t*-test was applied to compare the 150PE and 250PE sequencing read lengths. All differences were considered significant when *P* < 0.05.

## RESULTS

### DNA isolation, Illumina library preparation, and sequencing results

DNA extraction from the composite terrestrial soil and marine sediment mixture yielded a total of 6.2 µg DNA. Yield and DNA fragment size were sufficient for evaluating multiple SRS library preparation kits, sequencing lengths, and LRS.

Library yield and size distribution were assessed to compare SRS kit performance. Each SRS kit showed characteristic library yields and size distributions (Fig. S1). At 1 ng DNA input, the Ovation kit produced the highest library yield, followed by Nextera and NEB. At 100 ng DNA input, TruSeq resulted in a narrower library fragment distribution, followed by Ovation and NEB. Following the manufacturer’s recommendation, for 1 ng of DNA input, the NEB kit required fewer PCR cycles than Ovation and Nextera. This mechanistically accounts for the higher PCR duplication rates and library yields observed in the Ovation and Nextera data sets. Of note, the fewest PCR duplicates were generated with the TruSeq kit (Table 2), even though this kit did not require the lowest PCR cycles (Fig. S1).

**TABLE 2:** SRS Illumina sequencing results per replicate^*a*^^,^^*b*^

	NEB_1	NEB_100	NEXT_1	Ovation_1	Ovation_100	TruSeq_100	ANOVA
Total output reads (in million)							
150PE	50.5 ± 6.5a	26.8 ± 2.2b	42.5 ± 4.4a	34.4 ± 1.6c	54.4 ± 1.0a	16.2 ± 2.9d	L
250PE	50.4 ± 5.9a	56.9 ± 1.3a#	29.4 ± 0.6b#	37.0 ± 4.6c	40.2 ± 1.4c#	33.6 ± 2.1b#	L
Adapter content (%)							
150PE	34.0 ± 7.2a	28.3 ± 10.4a	1.5 ± 0b	2.5 ± 0b	2.5 ± 0b	0b	L
250PE	74.0 ± 5.3a#	72.7 ± 5.7a#	19.7 ± 1.5b#	42.3 ± 2.1c#	41.7 ± 1.5c#	2.3 ± 0.6a#	L
Average read length (bp)							
150PE	132 ± 2a	133 ± 7a	140 ± 2b,a	144 ± 1b	143 ± 1b	143 ± 1b	L
250PE	158.0 ± 9.3a#	161.8 ± 8.8a#	199.8 ± 18.5b#	203.8 ± 2.4b#	203.2 ± 1.0b#	223.3 ± 7.2b#	L
Duplicates (%)							
150PE	5.9 ± 0.8a	4.5 ± 0.7a	11.0 ± 0.8b	15.3 ± 0.7c	5.9 ± 0.1a	1.6 ± 0.3d	L
250PE	5.0 ± 1.0a	2.6 ± 0.4b#	4.8 ± 0.0a#	10.2 ± 1.0c#	2.9 ± 0.1b#	0.5 ± 0.1d#	L
GC content (%)							
150PE	61.7 ± 0.3a	61.3 ± 0.3a	61.0 ± 0.0b,a	61.5 ± 0.0a	61.7 ± 0.3a	62.0 ± 0.0c	L
250PE	65.3 ± 1.5a#	63.5 ± 1.3a	60.2 ± 0.3b#	61.5 ± 0.0b,a	61.3 ± 0.3b,a	61.2 ± 0.3b,a#	L

^*a*^ Total output reads, adapter content, average read length, duplicate sequences, and GC content of the different Illumina library preparation kits sequenced by 150PE or 250PE read-length mode.

^*b*^ Parameters were computed using FastQC v0.11.9 and MultiQC v1.10.1 tools. Results represent mean ± SEM (*n* = 3). Statistics: L, effect of the type of library preparation (one-way ANOVA, *P* < 0.05, and indicated when different). Values not sharing a common letter (a, b, c, and d) are significantly different (one-way ANOVA, *P* < 0.05). Letter “a” is used to indicate the first values. #, different vs 150PE group (Student’s *t*-test, *P* < 0.05).

Expectedly, libraries with shorter fragment sizes showed higher adapter content, particularly in the 250PE run, where longer read lengths increased the likelihood of sequencing into the adapters (Table 2).

After adapter trimming and quality filtering, the 150PE data set indicated that NEB libraries had an average effective insert length ~10 bp shorter than those prepared with the other kits, for both the 1 and 100 ng DNA inputs. For the 250PE run, TruSeq achieved the longest average effective insert length (223 bp), followed by Ovation (203 bp for both DNA inputs), Nextera (199 bp), and NEB (158–162 bp) (Table 2).

A consistent and statistically significant decrease in PCR duplicate rates was observed across all kits at 250PE compared to 150PE, suggesting that longer read lengths improve library complexity independently of kit chemistry.

### Impact of sequencing read length on assembly metrics

**Fig 2:**
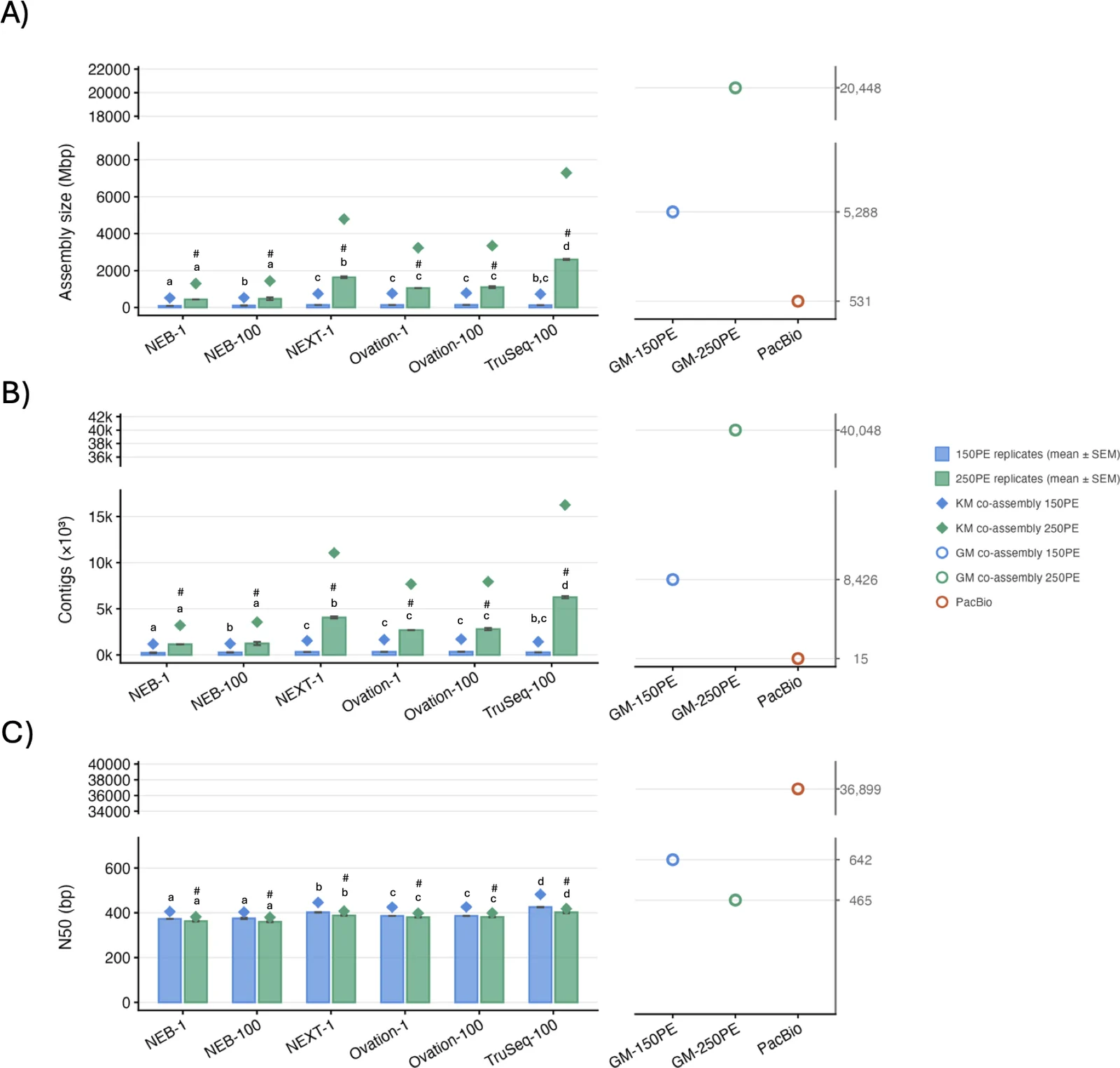
Comparison of library preparation methods and sequencing technologies for assembly metrics. Left panels show results for each Illumina library preparation kit at 150 bp paired-end (150PE, blue bars) and 250 bp paired-end (250PE, green bars) sequencing modes. Diamonds represent KM co-assembly values, and bars represent mean ± SEM (*n* = 3). Right panels show global-merge co-assembly values at 150PE (blue open circle) and 250PE (green open circle) alongside PacBio HiFi long-read sequencing (red open circle), with corresponding numbers on the right *y*-axis. A broken *y*-axis is used where indicated to accommodate the large range of values. (**A**) Total assembly size (Mbp). (**B**) Total number of contigs (×10^3^). (**C**) N50 (bp), representing the sequence length of the shortest contig at 50% of the total assembly length. Assembly metrics were calculated using assembly-stats v1.0.1 (sanger-pathogens/assembly-stats). Statistics for per-replicate data: letters indicate significant differences among library preparation kits (one-way ANOVA, *P* < 0.05); groups not sharing a letter differ significantly; # indicates significant differences between 150PE and 250PE within the same kit (Student’s *t*-test, *P* < 0.05).

For the SRS data sets, after trimming and subsampling, *de novo* assemblies were generated for each replicate, and assembly metrics were compared (Fig. 2; Table S1). Overall, NEB libraries showed the lowest N50, N90, and average contig lengths, while TruSeq and Nextera showed the highest values. The 250PE run showed significant improvements in all assembly metrics, including contig counts and gene recovery when compared to the 150PE sequencing mode (Fig. 2; Table S1), particularly for TruSeq and Nextera libraries, which showed the larger effective insert sizes.

Assembly metrics revealed that the GM co-assembly produced more contigs and larger assembly sizes than any of the single KM assemblies (Fig. 2; Table S1c) due to the higher sequencing depth. At 150PE, the GM assembly generated approximately 5.9× more contigs and 7.2× more assembled bases than the best-performing individual KM (TruSeq-150PE), while at 250PE, these differences were approximately 2.5× and 2.8×, respectively. The PacBio long reads further improved assembly stats, in particular contig sizes, with an average contig length of 35,894 bp and a largest contig of 8.27 Mbp, approximately 80× and 90× larger than those obtained with the best short-read KM assembly (TruSeq-250PE), respectively (Table S1c; Fig. 2).

TruSeq-250PE achieved the lowest cost per gigabase of usable sequencing data after quality trimming ($63.4/Gb), thus achieving a 3.5-fold reduction in cost per gigabase relative to PacBio HiFi ($219.2/Gb) (Table 1; Fig. 6).

### Library preparation and sequencing technologies affect taxonomy and diversity

**Fig 3:**
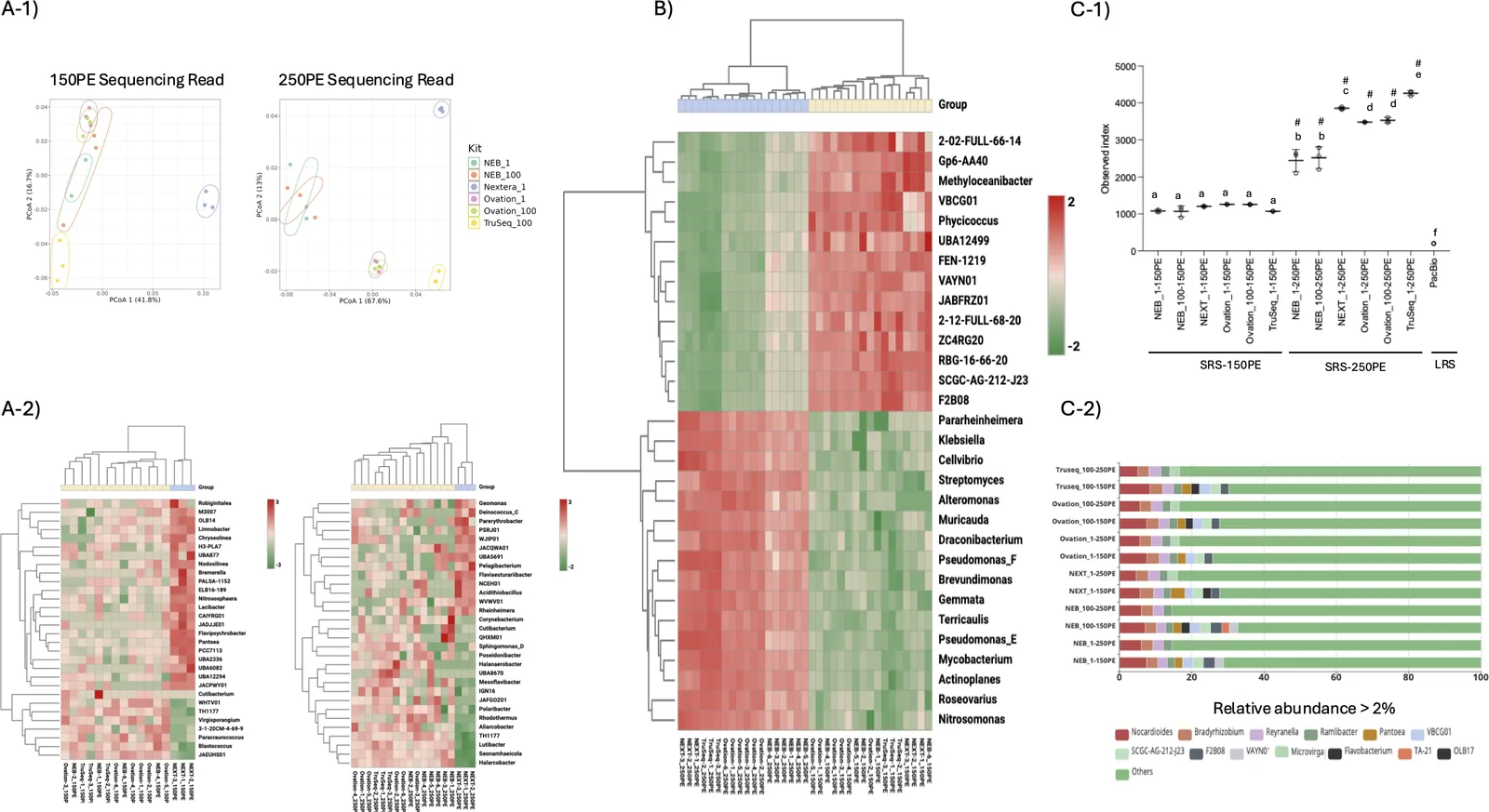
Comparison of library preparation kits and sequencing technologies for taxonomic assignment. (**A**-1) PCoA based on Bray-Curtis dissimilarity of detected OTUs for each Illumina library preparation condition and DNA input replicate. Each point represents a single sample and is colored by library preparation kit. Differences in community composition among groups were tested by PERMANOVA (*R*^2^ and *P*-value; 999 permutations). (**A**-2) Heatmap of the 10 most affected genera in Nextera libraries compared with the other kits, shown for both read lengths. Abundance is displayed as a color gradient. Hierarchical clustering is shown by the dendrograms on the left (OTUs) and top (samples) using Euclidean distance. Top annotation bars indicate experimental conditions; OTU names are shown on the right. (**B**) Heatmap of the top 30 affected taxa between the two read lengths, displayed as in panel **A**-2. (**C**-1) Alpha diversity (observed richness) at the genus level for each Illumina SRS kit and the PacBio LRS sample. Values are mean ± SEM (*n* = 3). Statistics: letters indicate differences among library preparation kits (one-way ANOVA, *P* < 0.05); groups not sharing a letter differ significantly; # indicates differences between 150PE and 250PE (Student’s *t*-test, *P* < 0.05). (**C**-2) Stacked bar plot of genus-level relative abundance community composition across Illumina kits and sequencing technologies. Percentages of total classified contigs are shown in parentheses. Genera with <2% relative abundance are grouped as “Others.”

Principal coordinate analysis of the OTUs at genus level revealed a significant difference between library preparation kits for both SRS runs, 150PE (adonis2 test, *R*^²^ = 0.617, *P* = 0.001) and 250PE (adonis2 test, *R*^²^ = 0.738, *P* = 0.001). Nextera libraries formed the most distinct clusters, indicating the largest differences in beta diversity associated with this kit (Fig. 3A-1). However, these trends were not statistically significant when tested across all kits using pairwise comparisons (data not shown). To further explore these patterns in ordination, a differential abundance analysis was performed comparing the 150PE and 250PE libraries. We identified 16 (11 overrepresented and 5 underrepresented) and 12 (2 overrepresented and 10 underrepresented) genera in Nextera libraries, respectively, when compared to other kits (FDR < 0.05 and a logFC ±1), with the top 10 affected taxa shown in Fig. 3A-2. Analysis of the genomic GC content of these differentially abundant taxa revealed a significant positive correlation between GC content and fold change at 150PE (Spearman *ρ* = +0.615, *P* = 0.033), with overrepresented genera showing a mean GC of 68.8% compared to 48.4% for underrepresented genera, while no significant correlation was observed at 250PE (*ρ* = −0.049, *P* = 0.880).

Next, a general differential abundance analysis comparison at the genus level of the two sequencing lengths (150PE vs 250PE) revealed that out of 5,301 genera meeting the criteria of more than 10 CPM, 775 genera were significantly affected (FDR < 0.05 and logFC ±1), with 633 overrepresented and 142 underrepresented in the 250PE data set. Hierarchical clustering of the top 30 affected taxa showed a clear separation between sequencing lengths, highlighting consistent behavior between the two SRS read lengths (Fig. 3B).

Finally, we compared the observed genera across all samples. The number of detected genera increased by 2.3-fold with NEB and by 4-fold with TruSeq when using 250PE reads compared to 150PE reads (Fig. 3C-1), supporting the differential abundance findings between the two sequencing lengths. Additionally, the relative abundance profiles showed a greater proportion of genera classified as “other” with the 250PE sequencing, indicating an increase in taxonomic diversity, especially for the rare and low-abundance genera (Fig. 3C-2). As expected, given its lower total read count relative to the SRS data sets, the PacBio library exhibited the lowest detected genus-level diversity (Fig. 3C-1), reflecting depth-dependent sampling rather than a biological difference in community composition.

### Assessment of protein prediction and annotation

**Fig 4:**
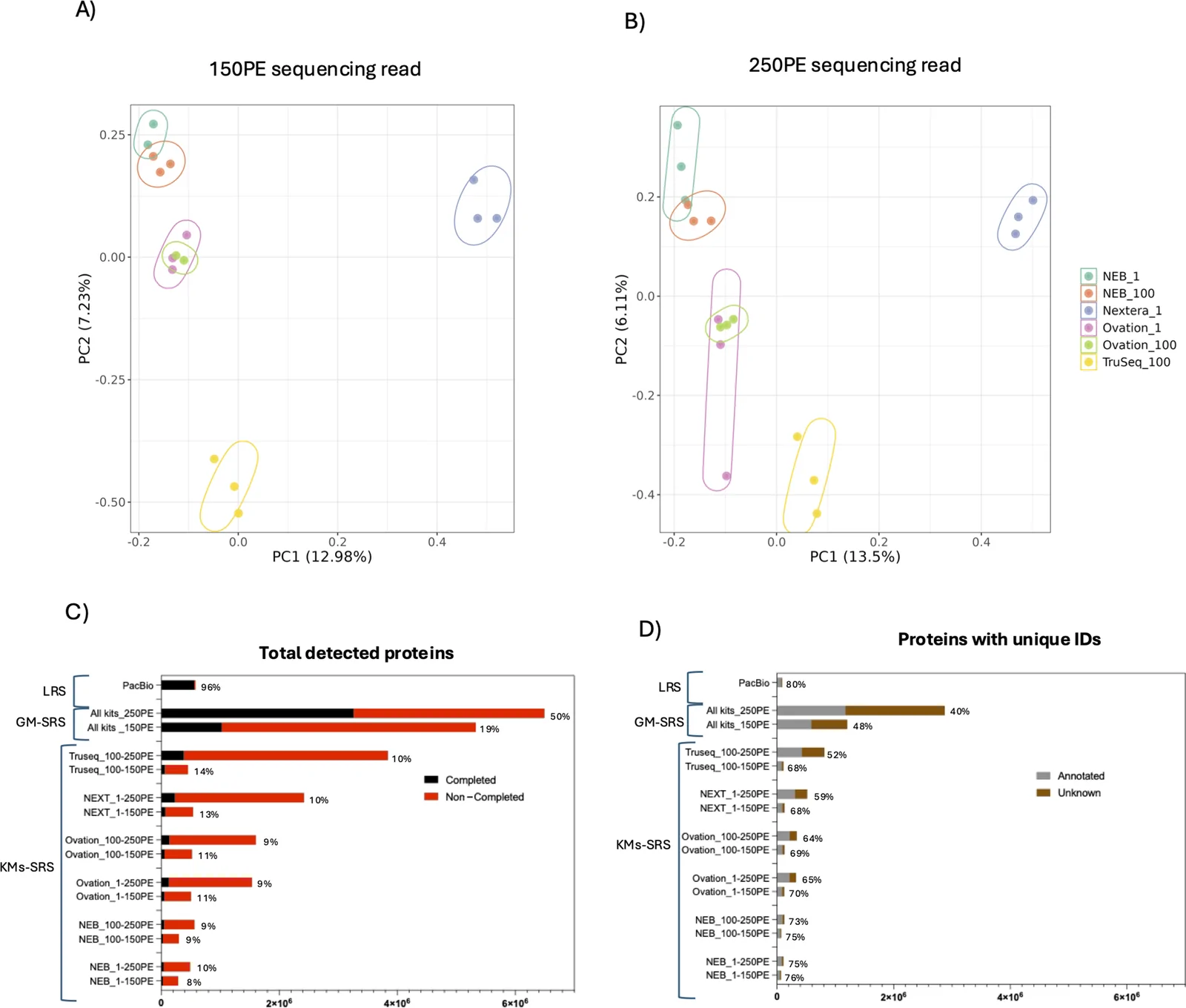
Comparison of library preparation kits and sequencing technologies for protein recovery. (**A and B**) PCoA based on Bray-Curtis dissimilarity of detected gene profiles for each Illumina library preparation method and DNA input replicate in the 150PE and 250PE runs. Each point represents a single sample and is colored by library preparation kit. Differences among groups were tested by PERMANOVA (*R*^2^ and *P*-value; 999 permutations). (**C**) Bar plot showing the total number of detected genes (completed and fragmented) across the different co-assemblies (KMs and GM) and sequencing technologies (Illumina SRS at different read lengths and PacBio HiFi LRS). Fragmented proteins were excluded to reduce artifacts; only contigs > 500 bp and proteins > 100 bp (33 amino acids) were retained. Values indicate the percentage of complete proteins relative to the total number of detected proteins. (**D**) Bar plot showing the number of unique proteins (annotated and unknown). Values indicate the percentage of annotated proteins relative to the total number of unique proteins.

Principal coordinate analysis of beta diversity revealed a significant difference across NGS library preparation kits (Fig. 4A and B) for both 150PE (adonis2, *R*^2^ = 0.371, *P* = 0.001) and 250PE (adonis2, *R*^2^ = 0.322, *P* = 0.001) runs in the total detected proteins per replicate. No significant difference in beta diversity was detected in the NEB and Ovation kits, regardless of the DNA input amount used for library preparation (data not shown).

For each KM, in the 150PE mode, we observed only small differences in the total number of predicted proteins identified across the various kits, as shown in Fig. 4C. In contrast, the 250PE mode significantly enhanced protein recovery and gene completeness, a result consistent with the assembly metrics reported in Table S1a. The TruSeq kit, in particular, showed the most substantial improvement, with protein recovery increasing nearly sevenfold. It also allowed the detection of approximately half of the non-completed proteins and around 10% of the completed ones when we compared against the GM catalog (with six times higher sequencing depth). The rest of the kits also showed performance that reflected the assembly statistics listed in Table S1c and the contig distribution in Fig. S2, with the protein detection worsening with the decline in assembly and contig quality.

In the case of the LRS data set, the total number of detected genes was in the range of the 150PE assemblies. However, the length of the assemblies allowed the retrieval of the complete sequence of 96% of the recovered genes. When analyzing the number of uniquely annotated proteins, in the 150PE run mode, we did not observe strong differences across the kits (Fig. 4D). In the GM, we observed a sharp increase in the total number of unique protein IDs, as expected due to the sixfold increase in sequencing depth. However, the same libraries run at 250PE showed a significant enrichment in the total number of unique proteins detected, especially for the kits with better assembly parameters, such as TruSeq and Nextera, followed by the Ovations. Additionally, for the TruSeq libraries, the number of unknown proteins increased from 34,990 in the 300 bp libraries to 389,567 in the 500 bp libraries at the same sequencing depth, representing more than a 10-fold increase. Finally, for the GM at 250PE, the number of unknown proteins had the highest relative increase. For the LRS, the total number of unique proteins was in the same range as the 300 bp KM data sets, although the percentage of the unknown proteins was generally lower.

### Metagenome-assembled genomes

**Fig 5:**
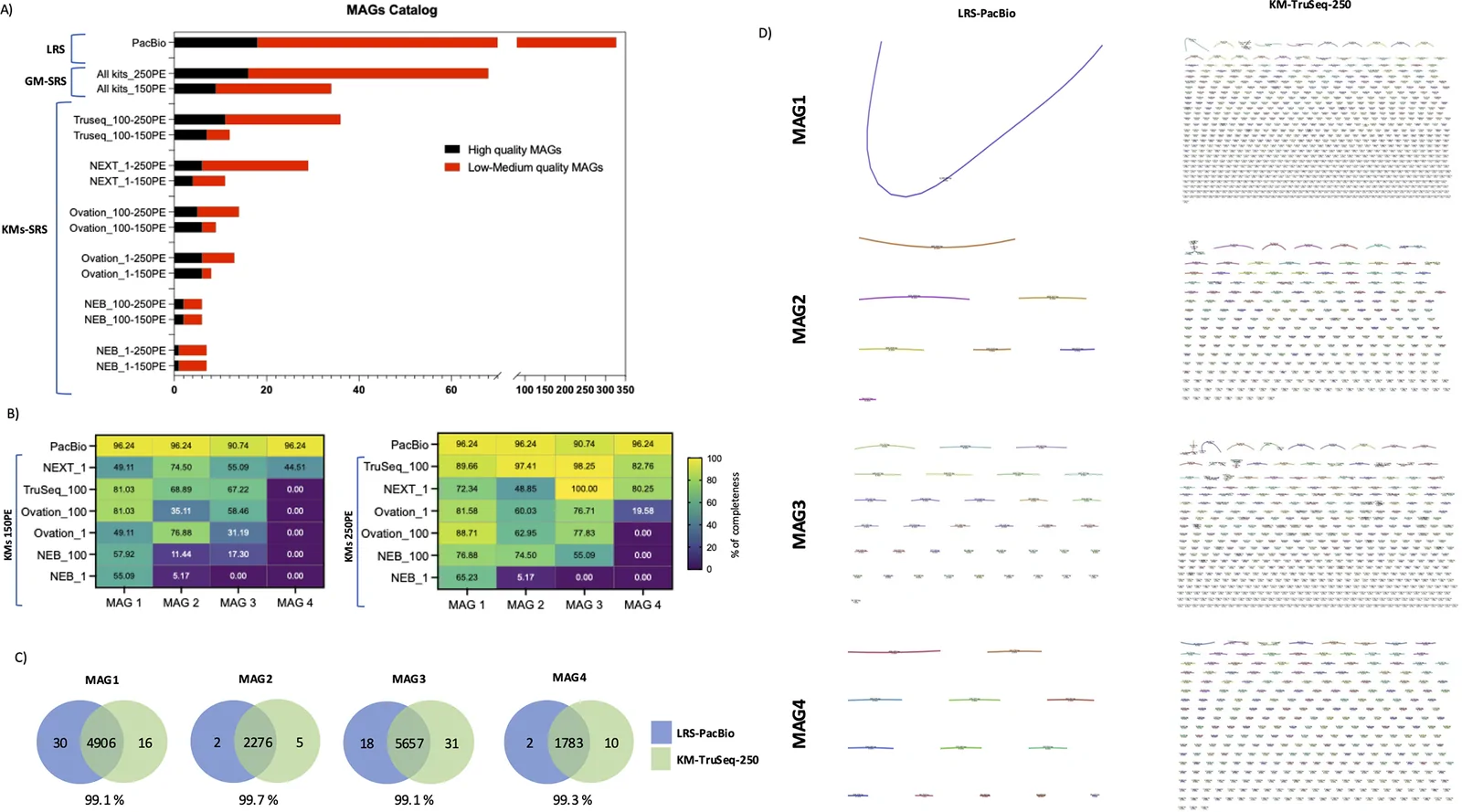
Comparison of library preparation kits and sequencing technologies for the MAG catalog. (**A**) Bar plot showing the total number of recovered MAGs (high and low-medium quality) across co-assemblies (KMs and GM) and sequencing technologies (Illumina SRS at different read lengths and PacBio HiFi LRS). MAG quality thresholds were defined as follows: low quality: completeness < 50% and contamination < 10%; medium quality: completeness > 50% and contamination < 10%; and high quality: completeness > 90% and contamination < 5%. (**B**) Heatmap showing MAG completeness (CheckM) for four new MAGs in KM Illumina libraries compared to PacBio. (**C**) Comparison of orthologous protein clusters among sequencing strategies using OrthoVenn3. The diagram displays the number of unique and shared orthologous clusters identified in MAGs recovered from Illumina SRS KM-TruSeq-250PE and PacBio HiFi LRS. Shared clusters represent conserved functions across data sets, while unique clusters may reflect method-specific functional recovery or assembly biases. OrthoVenn3 was run with default parameters (*E*-value cutoff, 1e-5; inflation value, 1.5). (**D**) Assembly graph representations for the four new MAGs from Illumina SRS KM-TruSeq-250PE and PacBio HiFi LRS technologies generated by Bandage.

We were also interested in measuring the effect of SRS vs LRS on MAG recovery. As shown in Fig. 5A, LRS produced substantially more MAGs than either GM or KM assemblies despite lower sequencing depth, resulting in more contiguous and accurate genome reconstructions. Shorter library inserts consistently yielded fewer and lower-quality MAGs across SRS kits, with MAG absolute recovery and quality highest in the TruSeq-250PE and Nextera-250PE KM combinations. The LRS-derived MAG results were obtained using 40- and 13.7-fold less data than the GM and the KM-TruSeq-250PE, respectively (Table S1c). The number of high-quality MAGs was in the same order of magnitude among the three data sets, ranging from 18 in the PacBio to 16 in the GM and 11 in the KM-TruSeq-250PE (Fig. 5A).

### Characterization of novel MAGs matching speciation threshold

The combination of the results from CheckM and GTDB-Tk in the LRS data set identified four high-quality MAGs that showed average nucleotide identity values below 95% against their closest GTDB reference genomes, which represents the standard speciation threshold (48). These results suggested that these MAGs could be potential novel species. We then set out to verify whether the same novel lineages were recoverable using the short-read strategies benchmarked in this study.

We ran a phylogenetic analysis of these new highest-quality LRS bins to describe them taxonomically (Fig. S3). These included (i) g__JABFRZ01 (f__GCA-002686595, *Planctomycetota*), (ii) g__VAYN01 (f__70-9, *Actinobacteriota*), (iii) an unclassified genus within *Pirellulaceae* family (*Planctomycetota*), and (iv) g__UBA11591 (f__*Vicingaceae*, *Bacteroidota*). All four MAGs showed classification only at high or intermediate taxonomic ranks (family or above), with placeholder genus names or no genus assignment at all. To further support the novelty of these four MAGs, we performed genome-based species delineation using TYGS. All four MAGs were classified as potential new species, with dDDH values (formula d4) against their closest type strains well below the 70% species delineation threshold: g__JABFRZ01 (closest type strain, *Caldalkalibacillus thermarum* and *Firmicutes*; dDDH, 25.0%), g__VAYN01 (closest type strain, *Caldalkalibacillus thermarum* and *Firmicutes*; dDDH, 26.8%), unclassified *Pirellulaceae* (closest type strain, *Candidatus Laterigemmans baculatus* and *Planctomycetota*; dDDH, 25.4%), and g__UBA11591 (closest type strain, *Candidatus Sulfidibacterium hydrothermale* and *Bacteroidota*; dDDH, 31.1%). The cross-phylum assignment of the closest type strain for g__JABFRZ01 and g__VAYN01 reflects the extreme phylogenetic divergence of these MAGs from all currently characterized lineages, further underscoring their novelty. These results are consistent with the ANI and phylogenetic analyses described above, collectively confirming that these MAGs represent genomically novel bacterial lineages.

Then, we calculated the individual percentage of ANI and the MASH distance between the LRS and the respective KM bins (Table S2) to verify the presence of similar bins in the SRS KMs. Once identified, we compared their percentage of completeness (Fig. 5B) against the LRS data. The KM-TruSeq-250PE combination achieved the highest completeness percentages and showed performance metrics most comparable to those obtained with the LRS approach (Tables S1d and S2). Overall, the long-read strategy produced better metrics and quality for these four MAGs, even though the total assembly data used (Table S1c) were more than 10 times larger in the KM-TruSeq-250PE. The number of contigs associated with each MAG was extremely different between the two technologies, showing high fragmentation in the KM-TruSeq-250PE. As Fig. 5D and Table S1d show, the number of contigs ranged from 1 to 37 in LRS, whereas in the SRS KM-TruSeq-250PE, it spanned from 279 to 833. Despite the high fragmentation observed in the KM-TruSeq-250PE SRS bins, OrthoVenn analysis revealed a strong overlap in orthologous clusters between KM-TruSeq-250PE SRS and PacBio LRS MAGs, with similarity values ranging from 99.1% to 99.7% (Fig. 5C). Our functional analysis with AntiSMASH 8.0 identified 38 biosynthetic gene clusters in the PacBio LRS and 46 in the KM-TruSeq-250PE SRS bins, including common types such as NRPS, Type I PKS, and terpenes. Several clusters had no close match in the MIBiG database (Table S3).

### Cost-efficiency analysis

**Fig 6:**
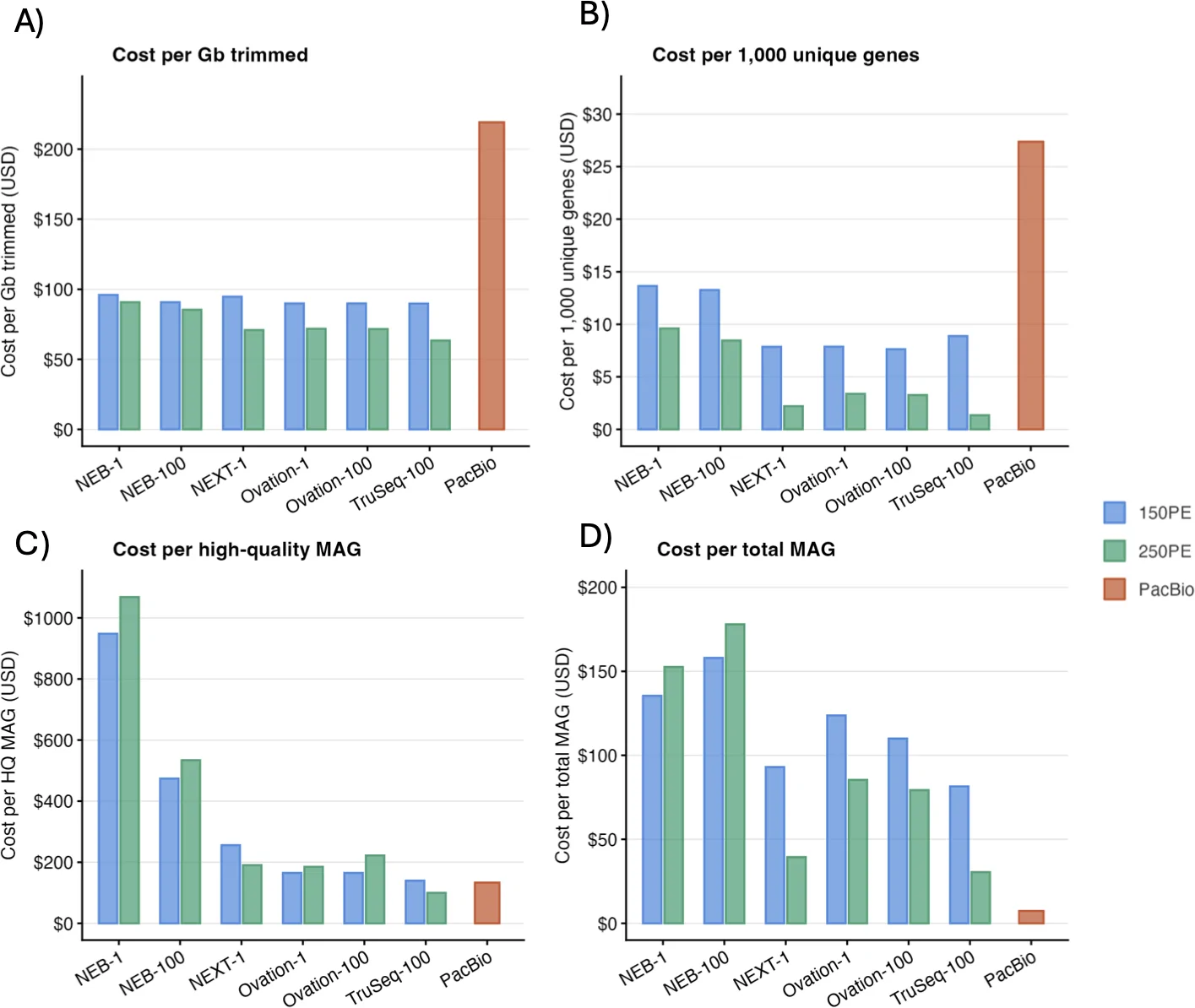
Cost-efficiency analysis of Illumina library preparation methods and PacBio HiFi long-read sequencing across four biological output metrics. Paired bars represent 150 bp paired-end (150PE, blue) and 250 bp paired-end (250PE, green) sequencing modes for each Illumina library preparation method. The PacBio HiFi bar (coral) represents a single sequencing run. (**A**) Cost per gigabase of trimmed sequencing data, calculated by dividing the total cost per sample by the total bases retained after adapter trimming and quality filtering. (**B**) Cost per 1,000 unique genes identified from kit-merge co-assemblies. (**C**) Cost per high-quality metagenome-assembled genome (completeness ≥ 90% and contamination ≤ 5%). (**D**) Cost per total MAG (completeness ≥ 50% and contamination ≤ 10%). Total costs include library preparation reagent and sequencing costs (Table 1) in triplicate for each SRS KM co-assembly and as a single run for LRS. Costs are estimated based on reagent and sequencing prices at the KAUST BCL at the time the experiments were performed.

To incorporate the economic dimension, we performed a concise cost-effectiveness analysis across four biological output metrics to compare library preparation kits and sequencing platforms based on reagent and sequencing prices at the time the experiments were performed (Fig. 6). When normalized to sequencing yield, TruSeq-100 at 250PE was the most cost-efficient kit ($63.4 per gigabase [Gb] trimmed), representing a 1.5-fold improvement over the least efficient kit (NEB-1 at 150PE; $96.0 per Gb). PacBio HiFi sequencing was the most expensive per Gb trimmed ($219.2), approximately 3.5-fold higher than TruSeq-250PE. The cost advantage of Illumina 250PE sequencing was most pronounced for unique gene discovery: TruSeq-250PE achieved a cost of $1.35 per 1,000 unique genes, compared to $27.37 for PacBio HiFi, a 20-fold difference, reflecting the substantially larger gene catalog recovered by this kit. For high-quality MAG recovery, TruSeq-250PE ($99.8 per HQ MAG) and PacBio HiFi ($133.4 per HQ MAG) were broadly comparable, while NEB-1 performed poorly regardless of read length ($948 to $1,068 per HQ MAG). In contrast, when considering total MAG yield, long-read sequencing was markedly more cost-efficient ($7.35 per total MAG vs $30.50 for TruSeq-250PE), owing to the 327 MAGs recovered by PacBio HiFi.

## DISCUSSION

The present study sought to maximize data acquisition and expand biological understanding in metagenomic analyses of complex and diverse environmental samples. Our approach leveraged widely used library preparation kits and sequencing platforms, and we evaluated these methods with low DNA input, a common scenario for samples originating from extreme environments. Additionally, we utilized LRS to benchmark metagenome assemblies and bins as an internal reference, enabling us to evaluate short-read library preparation protocols directly on the community of interest without the need to assemble low-diversity or artificial mock samples. Consistent with prior reports on SRS library preparation (10, 12, 14), we observed kit-specific differences among the six evaluated conditions in library yield, fragment-size distribution, PCR duplication rates, and clustering efficiency.

Overall, our comparison of different SRS kits revealed that the choice of library kit significantly influenced sequencing and assembly metrics, as well as taxonomic results, protein identification, and MAG recovery. In some of the cases, these effects were more evident when we utilized the kits with the 250PE sequencing read length. We found that the results were considerably influenced by the fragment size distribution of the libraries generated by the kits, which determines the effective insert length available for sequencing. Mechanistically, fragment-size differences reflect the fragmentation chemistry used (physical shearing, enzymatic digestion, or transposase-mediated tagmentation), each imposing distinct, reproducible biases that affect assembly, binning, and annotation. Biologically, these biases manifest as altered adapter contamination, assembly quality, gene coverage, and MAG reconstruction. Furthermore, the tendency of SRS platforms to sequence preferentially shorter fragments reduced the amount of longer inserts present in the data (60). In addition, a consistent decrease in PCR duplicate rates was observed at 250PE across all kits, further supporting the improvement in library complexity at longer read lengths. This effect could be related to the increased length of the sequenced product revealing sequence diversity otherwise not explored, although we cannot exclude that reduced base quality toward the end of reads (especially read 2; [61]) could confound library complexity analysis.

In our opinion, the length of the effective insert size alone could not account for the differences observed between the kits. We confirmed this by bioinformatically constraining the downstream analysis to rely only on reads with lengths between 202 and 250 bp for NEB-100 replicates, thus matching the insert length of the TruSeq-250 kit, the best-performing library prep. Although this approach improved the assembly results compared to the original data, the computed metrics remained significantly lower than those of the TruSeq or Nextera kits at 250PE at the same sequencing depth (Table S4). Our analysis, though, does not exclude that by selecting longer fragments, we biased the composition of those sequences, for example, excluding genes with a specific base composition less amenable to fragmentation.

Most genomic DNA-based sequencing library preparation protocols include a non-enzymatic shearing step. This method aims at breaking down the genome in an unbiased manner, thus creating a uniform distribution of DNA fragments independent of the base composition or other DNA physical properties. Conversely, enzymatic fragmentation is biased since cut sites are never random and enzyme efficiency is susceptible to inhibitors. Consequently, physical shearing of DNA has remained the gold standard in sequencing. However, although it is efficient and consistent, it can be expensive and time-consuming. The method requires specialized instruments and consumables, adding significant cost and handling time (62). Although solutions for the plate-based method already exist, those are not easily amenable to automation, thus making large-scale sequencing projects impractical for small labs and labor-intensive for larger ventures. Alternative methods available include endonucleases and transposase-mediated fragmentation and tagging (“tagmentation”). When the endonuclease-based fragmentation was introduced, concerns were raised that it might exhibit cut-site preference bias, therefore introducing artifacts (63). However, advancements in commercial enzyme preparations addressed most of these issues using a cocktail of enzymes (14). Nonetheless, Tanaka et al. (64) described that endonuclease-treated libraries exhibit a substantially larger number of recurrent artifactual single nucleotide variants (SNVs)/indels compared to those created through physical shearing. In a similar study, Gregory et al. (65) concluded that NGS library molecules produced by enzymatic fragmentation are not representative of the original DNA and are a byproduct of the enzymatic process. The authors found this observation consistent across different enzymatic fragmentation-based library preparation methods from other companies, suggesting that this is an inherent feature of the currently available commercial kits. On the other hand, and consistent with observations related to enzyme-based fragmentation technologies, Gunasekera et al. (66) reported biases in coverage, GC content, and *de novo* assembly quality metrics when using tagmentation methods as well. In agreement with these findings, Nextera libraries showed a pronounced GC-dependent bias in differential abundance at 150PE but not at 250PE. This pattern may reflect improved recovery of diverse genomic regions with longer reads, which can mitigate the relative influence of fragmentation-associated GC bias on taxonomic profiles.

We recognize that library fragmentation could be optimized for each library kit and that in environmental samples, other aspects, such as the presence of humic acids and enzyme inhibitors, can affect the efficiency of any given kit at a different level. However, ascertaining those effects goes beyond the scope of our study, requiring a sophisticated experimental design, material, time, and funds.

In metagenomics analysis, *de novo* assembly is a critical step that will dictate the downstream genomics results, influencing the protein catalog as well as the quantity and quality of MAGs (67, 68). We used Megahit, which is a de Bruijn graph-based *de novo* assembler, that can lead to graphs that are complex to interpret since this method is affected by low or uneven coverage or sequencing errors, thus affecting the accuracy of contigs and scaffolds used for genome assembly (69). Sequencing bias at the library preparation level could be a significant problem for existing *de novo* assembly algorithms. De Bruijn-based assemblers such as Megahit use an average coverage cutoff threshold for contigs to prune out low-coverage regions, which often contain more errors, thus reducing the complexity of the underlying de Bruijn graph. It also significantly affects the effective length and number of contigs in the final assembly, impacting the results (27). We found that the percentage of high-quality MAGs is higher in the kits that use sonication compared to the DNA enzymatic fragmentation step. This is in accord with Gregory and Tanaka’s observations of higher error rates on SNVs/indels introduced by the enzymatic approach (64, 65). Technically, library preparation-level sequencing bias propagates through the de Bruijn graph assembly, amplifying coverage unevenness into graph complexity that assembly algorithms cannot fully resolve. Biologically, this results in higher contamination rates and fewer high-quality MAGs in enzymatic fragmentation data sets, a pattern directly confirmed in our results.

The superior performance of the TruSeq kit can be attributed to a combination of mechanistic factors beyond fragment size distribution. The method requires a physical shearing step that, as we have discussed previously, provides a more uniform DNA fragment distribution, better suited for SRS (62). The size selection step removes the shortest and longest fragments and improves the homogeneous clustering on the NGS platforms (70). In our study, the Ovation kits, whose protocol did not require a size selection step, performed worse than the TruSeq kits, suggesting that this process may positively impact the subsequent steps. TruSeq consistently exhibited the lowest PCR duplication rates across both read lengths, even without reducing PCR cycles, and its size-selection step (absent in the Ovation protocol despite equivalent physical shearing) independently improves flow-cell clustering efficiency, confirming that fragment size alone does not account for the performance differences observed.

While sequencing depth has been described as crucial for gene discovery in marine microbial metagenomes (71), our results suggest that read length may be an equally important (and more cost-effective) parameter. We observed significant improvements in assembly metrics at 250PE without increasing read number, consistent with the relationship between read length and assembly contiguity established in benchmarking studies (72, 73), suggesting that optimizing sequencing length is an effective strategy for discovering novel proteins and taxa in complex environments.

Beyond NGS, as expected, third-generation LRS provides better assemblies, resulting in the recovery of high-quality MAGs and a higher percentage of fully assembled genes even using 10 times less sequencing data (68, 74). However, the overall count of detected proteins was significantly lower, although assembling more data could certainly contribute to improving protein detection. It is currently impractical to compare the number of proteins detected by the KM-TruSeq-250PE to the one detected by PacBio since the depth of sequencing is largely in favor of the SRS method (17.31 vs 10.96 Gb after quality trimming, at $63.4 vs $219.2 per Gb, respectively). This is likely to change in the near future, as continuous improvements in the sequencing output and cost reductions of long-read technologies become available. Despite this, the completeness of the proteins detected by the NGS-based kit was only 40%, whereas LRS reached 80%. However, this higher completeness must be interpreted in context, reflecting the quality of assembled genes rather than community-wide coverage. It is well known that PacBio sequencing technology struggles with the recovery and assembly of organisms present at low abundance (15), meaning that while LRS produces more complete gene models for the organisms it assembles, it may miss a substantial fraction of the community entirely. This limitation can impact studies of highly fragmented and complex biomes such as soil (75) or studies aimed at characterizing the rare biosphere (76). Although using third-generation LRS for metagenomic experiments offers substantial advantages, particularly for genome assembly and MAG recovery, it is also important to consider the cost and the stringent sample requirements. These aspects become particularly critical for large-scale projects, especially in extreme biomes that include samples with scarce DNA and genome integrity. An interesting approach is to include long-read sequencing for a subset of samples within a project to generate an internal reference MAG catalog, enabling robust benchmarking of short-read assemblies on real environmental data (24). This strategy could provide a realistic framework for evaluating sequencing and assembly performance in complex and novel microbial communities and for guiding future efforts aimed at maximizing overall MAG recovery. The recovery of four MAGs classified exclusively at high taxonomic ranks, with no cultured representatives, further highlights the potential of long-read sequencing to access microbial dark matter, lineages that remain largely uncharacterized and inaccessible to short-read approaches alone (5). Furthermore, functional analysis revealed several biosynthetic gene clusters with no close match in the MIBiG database, suggesting the potential for novel natural product biosynthesis in these underexplored lineages.

Although third-generation LRS offers substantial advantages for genome assembly and MAG recovery, cost and sample requirements must also be considered. At the time our study was conducted, PacBio HiFi was approximately 2.2× more expensive per sample than TruSeq 2 × 250 bp. Although it provided unmatched cost-efficiency for total MAG yield, Illumina 250PE sequencing was markedly more cost-effective for gene discovery and high-quality genome recovery, making it the preferred strategy for large-scale metagenomic studies prioritizing functional diversity.

Our key benchmarking finding indicates that the Illumina TruSeq library preparation kit, when combined with a 250PE sequencing read length, can yield results that closely align with those obtained using long-read technologies for high-quality MAGs in a diverse environmental sample, while recovering almost 10-fold more unique proteins than PacBio HiFi. Despite the fragmentation typical of short-read assemblies, the conservation of orthologous clusters found in the MAGs with long-read assemblies indicates reliable recovery of key functional genes and pathways. Additionally, this combination (TruSeq and 250PE) provides the highest number of protein recoveries among the SRS kits, offering the most holistic view of community diversity and function, an outcome not achievable with other library preparation or sequencing strategies.

While our findings provide critical guidance for complex environmental metagenomics, it is important to consider how these results may not extrapolate to other sample types. The performance differences we observed across library preparation kits and sequencing lengths are likely driven by the high taxonomic complexity and diversity of our composite environmental sample. In lower-complexity metagenomic samples, such as human gut or host-associated microbiomes, which are expected to be less diverse and dominated by a relatively limited number of lineages, the impact of protocol choice on downstream outcomes is expected to be reduced (12, 21). We therefore recommend that researchers consider sample complexity as an important factor when selecting library preparation strategies, as the gains associated with TruSeq at 2 × 250 bp, while substantial in our study, may not be equally realized across all sample types and research contexts.

### Conclusion

To our knowledge, this study provides the first direct assessment of 2 × 150 bp vs 2 × 250 bp Illumina short-read metagenomic sequencing across multiple library preparation kits, using the same complex environmental sample on the NovaSeq 6000. The findings highlight that read length can substantially influence the resolution of metagenomic assemblies and the reconstruction of microbial genomes, providing a valuable reference for future large-scale metagenomic projects. Our study concludes that selecting the appropriate library preparation method, sequencing length, and technology is crucial for enhancing outcomes of metagenomic studies and deciphering unknown microbial information. The optimal approach depends on study objectives and practical constraints. Based on our results and experience, for complex metagenomic samples, TruSeq at 250PE is the gold standard for achieving the best outcomes; however, it requires ≥100 ng DNA input, an amount often limited in extreme environments, and access to sonication equipment, which adds cost and hands-on time. Ovation at 250PE represents a competitive choice for low-input samples (~1 ng) under the same sonication-based workflow. Nextera XT is a cost-effective, hands-on, and automation-friendly attractive option, though its tagmentation chemistry can be susceptible to inhibitors, and a prior DNA purification step is advisable for soil samples. Taxonomic biases observed in this study should also be considered when interpreting community composition results. NEB-based kits, while accommodating the broadest input range and lowest cost, showed the weakest overall assembly performance and could be an option for projects with low diversity focused on taxonomic profiling without assembly, or where price, labor, and automation are favored, trading data quality for greater scalability. Finally, PacBio HiFi long-read sequencing remains the reference technology for contig contiguity, genome completeness, and high-quality MAG recovery. However, its high DNA input requirements, cost, and lower protein detection could limit its scalability for ambitious environmental projects. Our study highlights the critical importance of rigorous laboratory practices, thorough library quality control, and the careful selection of appropriate methodologies to enhance the detection of proteins and MAGs. These factors are essential for ensuring data comparability across samples and studies, particularly in large consortium projects and meta-analyses. This is especially relevant for data sets generated by commercial sequencing facilities, where details of library preparation methods are often insufficiently reported by the authors (77, 78).

Overall, our findings suggest that some current library preparation and sequencing technologies still limit our ability to fully capture microbial information in certain environmental samples, particularly those from complex, unknown, and underexplored environments. This indicates that a vast amount of untapped genetic content remains hidden, paving the way for future discoveries. Importantly, re-sequencing existing samples using a longer sequencing read length becomes a viable option, saving setup costs and allowing continued deciphering of the dark matter in complex biomes without needing new and costly sampling experiments.

## Supplementary Material

Reviewer comments

## Data Availability

All sequencing reads were deposited in the ENA database under project accession number PRJEB50284.

## SUPPLEMENTAL MATERIAL

Figure S1 spectrum.00013-26-s0001.tiff Library preparation results.

Figure S2 spectrum.00013-26-s0002.pdf Contig length distribution.

Figure S3 spectrum.00013-26-s0003.pdf Phylogenetic tree for the LRS MAGs.

Supplemental legends spectrum.00013-26-s0004.docx Legends for Figures S1 to S3 and Tables S2 to S3.

Table S1 spectrum.00013-26-s0005.docx Detailed assembly and MAG quality metrics for all library preparation kits and sequencing technologies.

Table S2 spectrum.00013-26-s0006.xlsx MAG assembly and quality metrics comparison between Illumina SRS and PacBio HiFi assemblies.

Table S3 spectrum.00013-26-s0007.xlsx Biosynthetic gene clusters (BGCs) identified in novel MAGs from Illumina SRS KM-TruSeq-250PE and PacBio HiFi assemblies.

Table S4 spectrum.00013-26-s0008.docx Illumina assembly and gene results before and after filtering

OPEN PEER REVIEW reviewer-comments.pdf An accounting of the reviewer comments and feedback.

ASM does not own the copyrights to Supplemental Material that may be linked to, or accessed through, an article. The authors have granted ASM a non-exclusive, world-wide license to publish the Supplemental Material files. Please contact the corresponding author directly for reuse.
