# Supplementary material for: Comparative metagenomic assessment of Illumina-compatible library preparation methods, short-read lengths, and PacBio HiFi sequencing reveals differences in microbial and functional diversity recovery from a complex environmental sample
Source: Microbiol Spectr. 2026 Jul 27;14(9):e00013-26. doi: 10.1128/spectrum.00013-26 (PMC13532183; doi:10.1128/spectrum.00013-26)
Supplement: Supplementary file 1 [file reviewer-comments.pdf]

AMERICAN SOCIETY FOR MICROBIOLOGY | Microbiology Spectrum

COMPARATIVE METAGENOMIC ASSESSMENT OF ILLUMINA-COMPATIBLE LIBRARY PREPARATION METHODS, SHORT-READ LENGTHS, AND PACBIO HIFI SEQUENCING REVEALS DIFFERENCES IN MICROBIAL AND FUNCTIONAL DIVERSITY RECOVERY FROM A COMPLEX ENVIRONMENTAL SAMPLE

Rubén Díaz-Rúa, Daniela Drautz-Moses, Xiang Zhao, Sadhasivam Perumal, Luke Esau, Angel Angelov, Alexander Putra, Patrick Driguez, Ming Cheung, and Emanuele Palescandolo

Corresponding Author(s): Rubén Díaz-Rúa, King Abdullah University of Science and Technology

Review Timeline:

Submission Date:	January 5, 2026
Editorial Decision:	April 1, 2026
Revision Received:	May 20, 2026
Editorial Decision:	May 31, 2026
Revision Received:	June 17, 2026
Accepted:	June 17, 2026

Editor: Vanessa Varaljay

Reviewer(s): The reviewers have opted to remain anonymous.

Transaction Report:

(Note: With the exception of the correction of typographical or spelling errors that could be a source of ambiguity, letters and reports are not edited. The original formatting of letters and referee reports may not be reflected in this compilation.)

DOI: https://doi.org/10.1128/spectrum.00013-26

Re: Spectrum00013-26 (**COMPARATIVE METAGENOMIC ASSESSMENT OF SHORT- AND LONG-READ SEQUENCING TECHNOLOGIES REVEALS UNKNOWN MICROBIAL INFORMATION IN A COMPLEX ENVIRONMENTAL SAMPLE**)

Dear Dr. Rubén Díaz-Rúa:

Thank you for the privilege of reviewing your work. Below you will find my comments, instructions from the Spectrum editorial office, and the reviewer comments.

Please ensure that you carefully address the reviewers' comments and concerns in your revision. In particular, the revision should incorporate recent and appropriate peer-reviewed citations, clearly state that the study includes a comparison of different Illumina library preparation kits, and explicitly distinguish between mechanistic interpretations and biological implications of the data and results

Please return the manuscript within 60 days; if you cannot complete the modification within this time period, please contact me. If you do not wish to modify the manuscript and prefer to submit it to another journal, notify me immediately so that the manuscript may be formally withdrawn from consideration by Spectrum.

Revision Guidelines
To submit your modified manuscript, log into the submission site at https://spectrum.msubmit.net/cgi-bin/main.plex. Go to Author Tasks and click the appropriate manuscript title to begin. The information you entered when you first submitted the paper will be displayed; update this as necessary. Note the following requirements:

• Upload point-by-point responses to the issues raised by the reviewers in a file named "Response to Reviewers," NOT in your cover letter.
• Upload a compare copy of the manuscript (without figures) as a "Marked-Up Manuscript" file.
• Upload a clean .DOC/.DOCX version of the revised manuscript and remove the previous version.
• Each figure must be uploaded as a separate, editable, high-resolution file (TIFF or EPS preferred), and any multipanel figures must be assembled into one file.
• Any supplemental material intended for posting by ASM should be uploaded with their legends separate from the main manuscript. You can combine all supplemental material into one file (preferred) or split it into a maximum of 10 files with all associated legends included.

For complete guidelines on revision requirements, see our Submission and Review Process webpage. Submission of a paper that does not conform to guidelines may delay acceptance of your manuscript.

Data availability: ASM policy requires that data be available to the public upon online posting of the article, so please verify all links to sequence records, if present, and make sure that each number retrieves the full record of the data. If a new accession number is not linked or a link is broken, provide Spectrum production staff with the correct URL for the record. If the accession numbers for new data are not publicly accessible before the expected online posting of the article, publication may be delayed; please contact production staff (Spectrum@asmusa.org) immediately with the expected release date.

Publication Fees: For information on publication fees and which article types are subject to charges, visit our website. If your manuscript is accepted for publication and any fees apply, you will be contacted separately about payment during the production process; please follow the instructions in that e-mail. Arrangements for payment must be made before your article is published.

ASM Membership: Corresponding authors may join or renew ASM membership to obtain discounts on publication fees. Need to upgrade your membership level? Please contact Customer Service at Service@asmusa.org.

The ASM Journals program strives for constant improvement in our submission and publication process. Please tell us how we can improve your experience by taking this quick Author Survey.

Thank you for submitting your paper to Spectrum.

Sincerely,
Vanessa Varaljay
Editor
Microbiology Spectrum

Reviewer #2 (Comments for the Author):

The study by Díaz-Rúa et al., describes the sequencing of a composite sample of DNA extracts from mangrove sediments and palm tree soil with different Illumina library creation kits and sequencing strategies. The authors compared these results to long-read PacBio data from the same composite sample too. The study found several differences and concluded that the longer shorter reads (250X250bp) and the mechanically shearing DNA library approach give the best results in terms of diversity of gene/genome recovered, which were comparable (except for the N50) to the PacBio data. While the results are interesting, the study does not make it clear what results are novel or highly unanticipated, compared to what has been published previously in the peer-reviewed literature or advertised by Illumina. The study does not cite several recent studies that did similar studies of applying short and long read sequencing on the same sample (e.g. Orellana et al. on North Sea samples, and several human microbiome studies). Accordingly, the novelty of the study has not been made clear in the view of this reviewer. It also looks like the study is focused on the different Illumina kits rather than short vs. long reads; and thus, the title and abstract give a somewhat misleading view. Further, I think it is important for the authors to, at least discuss, how other types of samples could behave and how extrapolatable their results will be to other samples. Methods seem to be adequately described overall. Data is available, which is good.

Minor/specific points:
Line 23. Specify what this sample was and if replicates were also taken?
Line 25-28. I think the abstract lacks some actual data. How much was the improvement of the longer short-read vs. the shorter ones? How much is the cost difference with long-read PacBio sequencing in their hands at the time of the writing (e.g. "a two-fold higher cost" or something like this would suffice to mention)?
Lines 67-76 and elsewhere in the Introduction. Almost all references cited here are <5 years old, on a topic that is very hot currently. The authors need to update their Introduction by citing relevant more recent papers, and especially highlighting the novelty of their results compared to the results of these studies.
Line 112. I would not call this a mock sample -or "engineered sample" elsewhere- because its composition is essentially unknown. A "composite sample of mangrove sediment and palm tree soil" is adequate (and subsequently, just call it "the sample"). Although each one of this two types of samples should be enough complex on its own; so it is not clear to me why the authors pool these extractions together. Was there any specific reason for this?
Lines 272-295. Are the results reported in the 1st paragraph of the Results new really or just repeat what Illumina advertise? The authors need to specifically highlight the novel results of their study.
Lines 303-309. By how much? Mention in the main text so that it stands independently from the figures or tables.
Line 319-321. Are there any characteristics of these differentially abundant taxa that could explain why they are differentially abundant, such as specific G+C% ranges? Such information could be useful to decide which kit gives more reliable results (in addition to the level of diversity recovered by each methodology, which is one important parameter).
Line 391. Against what? I think it is GTDB anchor genomes but this needs to be more clearly written, I think.
Lines 400-401. Taxonomic names should be in italics, here and elsewhere.
Lines 530-532. This has been adequately evaluated based on the CAMI challenge studies; please check their two publications.
lnes 540-542. With the same/similar sequencing effort (or similar cost instead per Gbp instead, for example)? The authors need to specify this within the text and should be reported after read quality trimming, I think.
Lines 588-592. Can the authors come up with specific suggestions (e.g., for which samples and objectives to use one methodology vs. the other) as opposed to these general statements? I think this would be more useful for readers.
Table 1. Add more results from Table 1 in the Results section such as the cost comparison?
I think most other tables could go to the supplement and instead have a figure with a couple panels in the main document that show the most important results of the tables (e.g., N50, number of contigs obtained) with bars/columns, which will make it easier to communicate the results.

Reviewer #3 (Comments for the Author):

Thank you for submitting this manuscript. The comparative assessment of library preparation kits and sequencing technologies is valuable, but several areas would benefit from clarification or refinement.

Comments:
The description of the environmental sample as "unique and highly diverse" would benefit from simplification. "Diverse" is sufficient, as "highly" is unquantified and "unique" applies to any combined samples.

Additional methodological detail is required regarding sample collection and handling. Please specify the collection sites, storage conditions, and whether samples were dried or otherwise processed or homogenised prior to DNA extraction.

Only one binning programme is used prior to dastool refinement. It would be helpful to explain this choice, particularly as relying on a single tool may omit bins that alternative programmes such as concoct or maxbin could recover and dastool could then combine. As is, it is possible that variation and differences are influenced by metabat2 clustering algorithms.

Version numbers should be provided for all software tools.

Taxonomic names should be italicised consistently throughout the manuscript.

The manuscript alternates between using the sample as a benchmarking resource and as a biological discovery dataset (e.g., the reporting of novel taxa and detection candidate gene families). The narrative would benefit from clearer distinction between these aims.

Please clarify whether a hybrid assembly was considered, as this could potentially improve completeness metrics.

Several conclusions would be strengthened by more mechanistic interpretation, particularly around why specific kits perform better or worse beyond fragment size distribution.

High quality MAGs should include full rRNA operons and sufficient tRNA representation. Please confirm whether these criteria were assessed alongside CheckM statistics.

The text could be made more concise. There are several sections where results are repeated across the main text, tables, and figures, and streamlining could possibly improve clarity.

Re: Spectrum00013-26 (**COMPARATIVE METAGENOMIC ASSESSMENT OF SHORT- AND LONG-READ SEQUENCING TECHNOLOGIES REVEALS UNKNOWN MICROBIAL INFORMATION IN A COMPLEX ENVIRONMENTAL SAMPLE**)

Editor Comments:

Thank you for the privilege of reviewing your work. Below you will find my comments, instructions from the Spectrum editorial office, and the reviewer comments.
Please ensure that you carefully address the reviewers' comments and concerns in your revision. In particular, the revision should incorporate recent and appropriate peer-reviewed citations, clearly state that the study includes a comparison of different Illumina library preparation kits, and explicitly distinguish between mechanistic interpretations and biological implications of the data and results.

Please return the manuscript within 60 days; if you cannot complete the modification within this time period, please contact me. If you do not wish to modify the manuscript and prefer to submit it to another journal, notify me immediately so that the manuscript may be formally withdrawn from consideration by Spectrum.

General response to the editor and all reviewers:
We would like to thank the editor and reviewers for their thoughtful and constructive evaluation of our manuscript. Their comments helped us to substantially improve the clarity, framing, and interpretation of our study.

In response, we have revised the title, running title, Abstract, Introduction, and Discussion to more clearly reflect the central focus of the manuscript: the comparative assessment of Illumina-compatible library preparation approaches, sequencing read length, and short- versus long-read sequencing technologies. We have also updated the Introduction and Discussion with recent peer-reviewed literature to better position the novelty of our study.

In addition, we have added quantitative comparisons throughout the Abstract, Results, and Discussion, including effects on assembly quality, protein recovery, MAG reconstruction, biosynthetic gene cluster detection, and sequencing cost efficiency. We have also clarified the nature and rationale of the composite environmental sample and expanded the Discussion to better distinguish mechanistic drivers of sequencing performance from their downstream biological implications.

In the following section, we provide a detailed point-by-point reply to each comment raised by the reviewers.

Reviewer #2:

The study by Díaz-Rúa et al., describes the sequencing of a composite sample of DNA extracts from mangrove sediments and palm tree soil with different Illumina library creation kits and sequencing strategies. The authors compared these results to long-read PacBio data from the same composite sample too. The study found several differences and concluded that the longer shorter reads (250X250bp) and the mechanically shearing DNA library approach give the best results in terms of diversity of gene/genome recovered, which were comparable (except for the N50) to the PacBio data.

1. While the results are interesting, the study does not make it clear what results are novel or highly unanticipated, compared to what has been published previously in the peer-reviewed literature or advertised by Illumina. The study does not cite several recent studies that did similar studies of applying short and long read sequencing on the same sample (e.g. Orellana et al. on North Sea samples, and several human microbiome studies). Accordingly, the novelty of the study has not been made clear in the view of this reviewer.

Response:

We thank the reviewer for this important and constructive critique. We fully agree that the novelty of our study was insufficiently articulated and that several relevant recent studies were not cited. We have addressed this concern in three ways in the revised manuscript.

First, we have added a new sentence to the Introduction that directly positions our work against the prior literature, including the studies the reviewer mentions, and highlights the key gaps our study addresses. Prior comparisons of short read length effects have relied on simulations or computational subsampling (Kang et al. 2022; Treiber et al. 2020) or different sequencing platforms (Sanders et al. 2019), and the only prior empirical suggestion of this effect appears in a manufacturer's application note (Illumina, M-NA-00010 v2.0) rather than the peer-reviewed literature. We note that this application note is a promotional manufacturer's document that has not undergone independent peer review, does not include statistical analysis, and compares only two library kits using a proprietary metagenomics pipeline (BaseSpace and DRAGEN); therefore, it does not constitute independent scientific evidence. The following sentence has been added to the Introduction (lines 122–130 in the revised manuscript):

"Current benchmarks in marine (Orellana et al. 2023) and host-associated environments (Chen et al. 2022; Gehrig et al. 2022; Eisenhofer et al. 2024) have significantly advanced our understanding of sequencing performance. However, these studies typically rely on a single library preparation kit and a fixed short-read length. While the Illumina application note ("Shotgun Metagenomics with Illumina DNA Prep on the NovaSeq 6000 System," n.d.)) compares multiple kits, it lacks long-read benchmarking, statistical validation, and functional assessments such as MAG discovery or biosynthetic potential. Crucially, no study to date has evaluated the combined effects of kit chemistry, read length, and DNA input concentration within a single complex environmental sample."

In addition, the following sentence has been revised in the final paragraph of the Introduction (lines 141–155 in the revised manuscript) to explicitly describe the experimental design addressing these gaps:

We systematically compared four short-read sequencing (SRS) library preparation kits encompassing three distinct chemistries, tested at one or two DNA input levels (1 and/or 100 ng) where the kit protocol permitted, and at two sequencing read lengths (2×150 bp and 2×250 bp). To benchmark against long-read sequencing (LRS), we processed the same DNA sample with PacBio HiFi, covering a total of thirteen distinct sequencing conditions."

Second, we have revised the Abstract, Importance, and Discussion to highlight protein discovery as a novel outcome metric. We show that TruSeq at 2×250 bp recovered more than sevenfold more unique proteins than the same kit at 2×150 bp (811,701 vs. 110,108), while recovering a comparable number of high-quality MAGs to PacBio HiFi (11 vs. 18) and surpassing it in protein discovery by almost tenfold (811,701 vs. 87,745) at less than half the sequencing cost. This dimension of benchmarking is not addressed in any prior short- vs. long-read benchmarking study. The following sentences have been added or revised in the Abstract (lines 31–35 in the revised manuscript), Importance (lines 54–59 in the revised manuscript), and Discussion (lines 862–866 in the revised manuscript):

Abstract: "*Notably, TruSeq libraries at 2×250 bp recovered more than sevenfold more unique proteins than the same kit at 2×150 bp (811,701 vs. 110,108) using the same number of sequencing reads, while recovering a comparable number of high-quality MAGs to PacBio HiFi long-read sequencing (11 vs. 18) and surpassing it in protein discovery by almost tenfold (811,701 vs. 87,745) at less than half of the sequencing cost*."

Importance: *"These findings demonstrate that short-read sequencing at 2×250 bp, with appropriate library preparation, can match long-read technologies in MAG recovery while substantially surpassing them in protein discovery. With less than half of the sequencing price and a 3.5-fold reduction in cost per gigabase of usable data, this method facilitates more accessible large-scale metagenomic analysis within complex environmental systems."*

Discussion: *"Our key benchmarking finding indicates that the TruSeq Illumina library preparation kit, when combined with a 250PE sequencing read length, can yield results that closely align with those obtained using long-read technologies for high-quality MAGs in a diverse environmental sample, while recovering almost tenfold more unique proteins than PacBio HiFi."*

Third, we have added BGC analysis of high-quality MAGs to the Abstract section, linking sequencing strategy to natural product discovery potential. The BGC analysis serves as a final biological validation of our sequencing strategy, confirming that the kit and read length choices made upstream translate into meaningful biological outcomes. This analysis specifically demonstrates that the optimized short-read strategy (TruSeq-250PE) delivers biosynthetic gene cluster reconstruction comparable to PacBio HiFi long-read sequencing, providing biological evidence that technical improvements in assembly quality have real downstream consequences for natural product discovery. The following sentence has been added to the Abstract (lines 35–37 in the revised manuscript):

"Furthermore, BGC analysis identified 46 biosynthetic gene clusters in TruSeq-250PE assemblies compared to 38 in PacBio HiFi, with several showing no close match in the MIBiG database."

2. It also looks like the study is focused on the different Illumina kits rather than short vs. long reads; and thus, the title and abstract give a somewhat misleading view.

Response:

We thank the reviewer for this observation and agree that the original title did not adequately reflect the full scope of the study. We have revised the title to explicitly include the comparison of Illumina-compatible library preparation kits alongside read lengths and long-read sequencing, providing a more accurate representation of the study design. The revised title now reads:

"COMPARATIVE METAGENOMIC ASSESSMENT OF ILLUMINA-COMPATIBLE LIBRARY PREPARATION METHODS, SHORT-READ LENGTHS, AND PACBIO HIFI REVEALS UNKNOWN MICROBIAL INFORMATION IN A COMPLEX ENVIRONMENTAL SAMPLE"

The running title has also been revised to:

" Library Preparation and Read Length Shape Metagenomics"

In addition, the Abstract has been revised to explicitly state that the study benchmarks six Illumina-compatible library preparation conditions encompassing three distinct chemistries alongside PacBio HiFi long-read sequencing, as detailed in our response to Comment 1 above.

3. Further, I think it is important for the authors to, at least discuss, how other types of samples could behave and how extrapolatable their results will be to other samples. Methods seem to be adequately described overall. Data is available, which is good.

Response:

We thank the reviewer for this important suggestion. We agree that discussing the extrapolability of our findings to other sample types is valuable for readers and strengthens the broader impact of the study.

One of our central arguments is that the performance differences observed across library preparation kits and sequencing lengths are largely driven by the high taxonomic complexity of our composite environmental sample, and that these effects may be less pronounced in lower-complexity matrices such as mock communities, host-associated or gut microbiomes. This is supported by Illumina's benchmarking data (Application Note M-NA-00010), which demonstrates that protocol-dependent performance differences are more detectable in high-diversity soil samples than in stool, as well as by published benchmarking studies in human microbiome contexts (Gaulke et al. 2021). The new paragraph reads (<u>lines 873–903 in the revised manuscript</u>):

"While our findings provide critical guidance for complex environmental metagenomics, it is important to consider how these results may not extrapolate to other sample types. The performance differences we observed across library preparation kits and sequencing lengths are likely driven by the high taxonomic complexity and diversity of our composite environmental sample. In lower-complexity metagenomic samples, such as human gut or host-associated microbiomes, which are expected to be less diverse and dominated by a relatively limited number of lineages, the impact of protocol choice on downstream outcomes is expected to be reduced ("Shotgun Metagenomics with Illumina DNA Prep on the NovaSeq 6000 System," n.d.; Gaulke et al. 2021). We therefore recommend that researchers consider sample complexity as an important factor when selecting library preparation strategies, as the gains associated with TruSeq at 2×250

bp, while substantial in our study, may not be equally realised across all sample types and research contexts."

Minor/specific points:

Line 23. Specify what this sample was and if replicates were also taken?

Response:
We thank the reviewer for this observation. We have revised the Abstract to specify that the sample consisted of a composite of marine mangrove sediment and terrestrial palm tree soil, and that libraries were prepared in triplicate. This information was present in the Materials and Methods section but was inadvertently omitted from the Abstract. The revised text now reads (lines 26-29 of revised manuscript):

"...at 2×150 bp and 2×250 bp read lengths alongside PacBio HiFi long-read sequencing using a composite environmental sample of marine mangrove sediment and terrestrial palm tree soil, with Illumina-compatible libraries prepared in triplicate."

Line 25-28. I think the abstract lacks some actual data. How much was the improvement of the longer short-read vs. the shorter ones? How much is the cost difference with long-read PacBio sequencing in their hands at the time of the writing (e.g. "a two-fold higher cost" or something like this would suffice to mention)?

Response:
We thank the reviewer for this observation. We have revised the Abstract to include specific quantitative data on the improvement achieved with longer short reads, and to specify the cost difference with PacBio HiFi long-read sequencing. The revised text now reads (lines 32-38 of revised manuscript):

"Notably, TruSeq libraries at 2×250 bp recovered more than sevenfold more unique proteins than the same kit at 2×150 bp (811,701 vs. 110,108) using the same number of sequencing reads, while recovering a comparable number of high-quality MAGs to PacBio HiFi long-read sequencing (11 vs. 18) and surpassing it in protein discovery by almost tenfold (811,701 vs. 87,745) at less than half of the sequencing cost."

In line with that, the cost comparison is further detailed in subsequent comments below, where we provide a comprehensive cost-efficiency analysis across all library preparation kits and sequencing conditions.

Lines 67-76 and elsewhere in the Introduction. Almost all references cited here are <5 years old, on a topic that is very hot currently. The authors need to update their Introduction by citing relevant more recent papers, and especially highlighting the novelty of their results compared to the results of these studies.

Response:

We thank the reviewer for this comment and have addressed it in two ways:

First, we have replaced outdated references with more recent peer-reviewed publications, preserving the original text in all cases:

- Berini et al. 2017 → Robinson et al. 2021 (line 83 in the revised manuscript)
- Trindade et al. 2015 → Paoli et al. 2022; Wiederkehr et al. 2026 (line 84 in the revised manuscript)
- Lorenz and Eck 2005 → Hogg et al. 2024 (lines 84-85 in the revised manuscript)
- Costea et al. 2017 → Tourlousse et al. 2021; Galla et al. 2024 (line 87-88 in the revised manuscript)
- Jones et al. 2015 → Gaulke et al. 2021 (line 88 in the revised manuscript)
- Chen et al. 2022 → Orellana et al. 2023 (line 89 in the revised manuscript)

Second, the explicit highlighting of our results against the prior literature, including the new positioning sentence added to the Introduction, has been addressed in detail in our response to Comment 1 above.

Line 112. I would not call this a mock sample -or "engineered sample" elsewhere- because its composition is essentially unknown. A "composite sample of mangrove sediment and palm tree soil" is adequate (and subsequently, just call it "the sample"). Although each one of this two types of samples should be enough complex on its own; so it is not clear to me why the authors pool these extractions together. Was there any specific reason for this?

Response:

We thank the reviewer for this comment and fully agree that the terms "mock" and "engineered sample" were inappropriate. We have replaced all instances throughout the manuscript with "composite sample of mangrove sediment and palm tree soil" at first mention and "the sample" subsequently.

Regarding the rationale for pooling, the strategy was deliberate and motivated by three considerations. First, previous benchmarking studies have shown that samples of insufficient microbial complexity fail to reveal meaningful differences across library preparation methods and sequencing strategies, as sequencing coverage becomes rapidly saturated in low-diversity communities (Sanders et al. 2019). This limitation is further illustrated by the contrast between stool and soil samples in Illumina's own benchmarking data (Illumina Application Note M-NA-00010): stool showed markedly less variation in sequencing performance metrics compared to soil, where higher microbial diversity allowed differences across platforms and protocols to become clearly detectable. Second, our sampling sites in Saudi Arabia are located in a hyper-arid region where extreme temperatures and aridity are known to constrain microbial diversity in both terrestrial and coastal ecosystems (Maestre et al. 2015). This concern is particularly relevant to our marine mangrove sampling site: Thomson et al. (2022), working in the Red Sea mangroves near Thuwal, the precise location of our sampling, documented reduced alpha diversity and a shift towards stress-tolerant taxa under the extreme summer conditions of high temperature and

hypersalinity that characterise this environment. Our samples were collected on 25 June 2021, during summer conditions with average temperatures of 36 to 40°C, when these diversity-reducing pressures would have been active. By combining DNA from mangrove sediment and palm tree soil, we deliberately broadened the taxonomic range captured, compensating for the potentially lower alpha diversity expected at each individual arid site. Third, pooling five DNA extractions per environment and combining them equimolarly minimised confounding variation arising from spatial heterogeneity and extraction batch effects, ensuring a homogeneous and reproducible specimen across all conditions tested, essential for rigorous cross-condition comparisons.

The following sentences have been revised and added to the Methods section (lines 171–178 in the revised manuscript):

"A diverse composite sample of mangrove sediment and palm tree soil was assembled to provide sufficient DNA quantity and quality for comprehensive testing across various library preparation kits and sequencing technologies without being influenced by different sampling sites and DNA extraction methods. This pooling strategy was adopted to ensure sufficient microbial complexity for discriminatory benchmarking, given that samples of low diversity have been shown to mask protocol-dependent differences (Sanders et al. 2019), and that arid environments such as our sampling sites are known to harbour reduced microbial diversity (Maestre et al. 2015; Thomson et al. 2022)."

Lines 272-295. Are the results reported in the 1st paragraph of the Results new really or just repeat what Illumina advertise? The authors need to specifically highlight the novel results of their study.

Response:

We thank the reviewer for this observation. We have removed the sentences describing clustering efficiency and data yield differences between kits, as these are well-characterised by Illumina. Although the remaining library preparation metrics are consistent with previously reported findings (Sato et al. 2019; Gaulke et al. 2021; Tourlousse et al. 2021), we have retained them as they are necessary to establish the mechanistic basis for the downstream differences in assembly quality, protein recovery, and MAG reconstruction. Upon closer examination of the data prompted by this comment, we identified a consistent and statistically significant decrease in PCR duplicate rates across all kits at 250PE compared to 150PE (a finding not previously reported), which has been added to the manuscript and discussed in the Discussion. Sato et al. 2019, has been added to the reference list.

The following sentences have been removed from the Results:

"We observed that shorter fragments (Ovation and NEB libraries) clustered more efficiently than the longer fragments of the Nextera and TruSeq libraries (Table 2). The latter produced three times less data in the 150PE run than the highest-yielding library despite equimolar loading concentrations. Nextera libraries displayed a size distribution similar to Ovation but with enrichment in longer fragments (Supplementary Figure 1)."

The following sentence has been added at the end of the sequencing outcomes paragraph in the Results (lines 407–409):

"Notably, a consistent and statistically significant decrease in PCR duplicate rates was observed across all kits at 250PE compared to 150PE, suggesting that longer read lengths improve library complexity independently of kit chemistry."

The following sentence has been added at the opening of the Overall paragraph in the Discussion (lines 659–662):

"Consistent with prior reports on SRS library preparation (Sato et al. 2019; Gaulke et al. 2021; Tourlousse et al. 2021), we observed kit-specific differences among the six evaluated conditions in library yield, fragment-size distribution, PCR duplication rates, and clustering efficiency."

The following sentence has been added to the same paragraph in the Discussion (lines 675–691):

" In addition, a consistent decrease in PCR duplicate rates was observed at 250PE across all kits, further supporting the improvement in library complexity at longer read lengths. This effect could be related to the increased length of the sequenced product revealing sequence diversity otherwise not explored, although we cannot exclude that reduced base quality toward the end of reads (especially read 2; (Tan et al. 2019)) could confound library complexity analysis."

Lines 303-309. By how much? Mention in the main text so that it stands independently from the figures or tables.

Response:

We thank the reviewer for this suggestion. We have revised the text to include specific quantitative values so that the results stand independently of Table 5 and Supplementary Figure 2. The revised text now reads (lines 431–442 of the revised manuscript):

"Assembly metrics revealed that the GM co-assembly produced more contigs and larger assembly sizes than any of the single KM assemblies (Table 5), due to the higher sequencing depth. At 150PE, the GM assembly generated approximately 5.9× more contigs and 7.2× more assembled bases than the best-performing individual KM (TruSeq-150PE), while at 250PE these differences were approximately 2.5× and 2.8×, respectively. The PacBio long reads further improved assembly stats, in particular contig sizes, with an average contig length of 35,894 bp and a largest contig of 8.27 Mbp, approximately 80× and 90× larger than those obtained with the best short-read KM assembly (TruSeq-250PE), respectively (Table 5 and Supplementary Figure 2)."

Line 319-321. Are there any characteristics of these differentially abundant taxa that could explain why they are differentially abundant, such as specific G+C% ranges? Such information could be useful to decide which kit gives more reliable results (in addition to the level of diversity recovered by each methodology, which is one important parameter).

Response:

We thank the reviewer for this valuable suggestion. We have now analysed the genomic GC content of all differentially abundant (DA) taxa identified in the Nextera vs. other kits comparison ($FDR < 0.05$, $|logFC| \geq 1$) and incorporated the results into both the Results and Discussion sections.

At 2×150 bp, a statistically significant positive correlation was found between GC content and fold change (Spearman $\rho = +0.615$, $p = 0.033$), with over-represented genera being exclusively high-GC organisms (mean GC 68.8%; Cutibacterium 60%, JAEUHS01 72%, Blastococcus 74%) compared to under-represented genera spanning a moderate GC range (mean GC 48.4%; 38 to 64%), consistent with the GC-dependent bias of tagmentation reported by Gunasekera et al. (2021). At 2×250 bp, no significant correlation was observed (Spearman $\rho = -0.049$, $p = 0.880$), a pattern that may reflect the improved assembly of a broader range of genomic regions enabled by longer reads, which could reduce the relative impact of fragmentation-associated GC bias on taxonomic recovery.

The following sentence has been added to the Results section (Library Preparation and Sequencing Technologies Affect Taxonomy and Diversity), lines 464–468:

"Analysis of the genomic GC content of these differential abundance taxa revealed a significant positive correlation between GC content and fold change at 150PE (Spearman $\rho = +0.615$, $p = 0.033$), with over-represented genera showing a mean GC of 68.8% compared to 48.4% for under-represented genera, while no significant correlation was observed at 250PE ($\rho = -0.049$, $p = 0.880$)."

The following sentence has been added to the Discussion (lines 727-734), replacing the original closing sentence:

"On the other hand, and consistent with observations related to enzyme-based fragmentation technologies, Gunasekera et al. reported biases in coverage, GC content, and de novo assembly quality metrics also when using tagmentation methods (Gunasekera et al. 2021). In agreement with these findings, Nextera libraries showed a pronounced GC-dependent bias in differential abundance at 150PE but not at 250PE. This pattern may reflect improved recovery of diverse genomic regions with longer reads, which can mitigate the relative influence of fragmentation-associated GC bias on taxonomic profiles."

Line 391. Against what? I think it is GTDB anchor genomes but this needs to be more clearly written, I think.

Response:
We thank the reviewer for this observation. The sentence has been revised to explicitly state that the ANI values were calculated against the closest GTDB reference genomes, making the comparison unambiguous. The original sentence reads:

"The combination of the results from CheckM and GTDB-Tk in the LRS dataset identified four high-quality MAGs that showed an average nucleotide identities (ANIs) value under 95%, which represents the standard speciation threshold (Jain et al. 2018)."

The revised sentence now reads (lines 552-555):

"The combination of the results from CheckM and GTDB-Tk in the LRS dataset identified four high-quality MAGs that showed average nucleotide identity (ANI) values below 95% against their closest GTDB reference genomes, which represents the standard speciation threshold (Jain et al. 2018)."

Lines 400-401. Taxonomic names should be in italics, here and elsewhere.

Response:
We thank the reviewer for this observation. We have systematically reviewed the entire manuscript and corrected all taxonomic names to italics throughout the text.

Regarding taxon names appearing within figures (e.g., heatmap row labels, phylogenetic tree tips), these have been italicized where the figure format permitted direct editing. In cases where names are rendered programmatically, and italicization is not supported by the plotting software, the corresponding names are correctly italicized in the associated figure legend.

Note: GTDB placeholder identifiers (e.g., g__JABFRZ01, f__GCA-002686595, g__VAYN01, g__UBA11591) are database identifiers, not formal taxonomic names, and have therfore not been italicised.

Lines 530-532. This has been adequately evaluated based on the CAMI challenge studies; please check their two publications.

Response:
We thank the reviewer for this suggestion. We have revised and condensed the paragraph below, incorporating citations to both CAMI challenge publications (Sczyrba et al. 2017; Meyer et al. 2022). We have revised this section as follows (lines 787-808 of the revised manuscript):

"While sequencing depth has been described as crucial for gene discovery in marine microbial metagenomes (Duarte et al. 2020), our results suggest that read length may be an equally important (and more cost-effective) parameter. We observed significant improvements in assembly metrics at 250PE without increasing read number, consistent with the relationship between read length and assembly contiguity established in benchmarking studies (Sczyrba et al. 2017; Meyer et al. 2022), suggesting that optimizing sequencing length is an effective strategy for novel protein and taxa discovery in complex environments."

Lines 540-542. With the same/similar sequencing effort (or similar cost instead per Gbp instead, for example)? The authors need to specify this within the text and should be reported after read quality trimming, I think.

Response:
We thank the reviewer for this suggestion. We agree that quantifying the sequencing effort difference after quality trimming adds important context to this comparison. We have revised the relevant sentence in the Discussion and added a sentence to the Results to include the actual Gbp and cost per Gbp values after trimming for both TruSeq-250PE and PacBio HiFi.

The following sentence has been added to the Results, Impact of Sequencing Read Length on Assembly Metrics section (lines 440–442 of the revised manuscript):

"Notably, TruSeq-250PE achieved the lowest cost per gigabase of usable sequencing data after quality trimming ($63.4/Gbp), thus achieving a 3.5-fold reduction in cost per gigabase relative to PacBio HiFi ($219.2/Gbp) (Table 1; Figure 6)."

The following revised sentence now appears in the Discussion (lines 815–816 of the revised manuscript):

"It is currently impractical to compare the number of proteins detected by the KM-TruSeq-250PE to the one detected by PacBio since the depth of sequencing is largely in favor of the SRS method (17.31 Gbp vs. 10.96 Gbp after quality trimming, at $63.4 vs. $219.2 per Gbp, respectively)."

Lines 588-592. Can the authors come up with specific suggestions (e.g., for which samples and objectives to use one methodology vs. the other) as opposed to these general statements? I think this would be more useful for readers.

Response:

We thank the reviewer for this constructive suggestion. We agree that the original text was too general to be of practical use to readers. The Conclusion has been revised to provide specific, evidence-based recommendations for each library preparation kit and sequencing strategy evaluated in this study, explicitly linking methodology selection to sample type, DNA input requirements, study objectives, and practical constraints.

The revised text now reads (lines 911–928 of the revised manuscript):

"The optimal approach depends on study objectives and practical constraints. Based on our results and experience, for complex metagenomic samples, TruSeq at 250PE is the gold standard for achieving the best outcomes; however, it requires ≥100 ng DNA input, an amount often limited in extreme environments, and access to sonication equipment, which adds cost and hands-on time. Ovation at 250PE represents a competitive choice for low-input samples (~1 ng) under the same sonication-based workflow. Nextera XT is a cost-effective, hands-on and automation-friendly attractive option, though its tagmentation chemistry can be susceptible to inhibitors, and a prior DNA purification step is advisable for soil samples. Taxonomic biases observed in this study should also be considered when interpreting community composition results. NEB-based kits, while accommodating the broadest input range and lowest cost, showed the weakest overall assembly performance and could be an option for projects with low diversity focused on taxonomic profiling without assembly, or where price, labor, and automation are favored, trading data quality for greater scalability. Finally, PacBio HiFi long-read sequencing remains the reference technology for contig contiguity, genome completeness, and high-quality MAG recovery. However, its high DNA input requirements, cost, and lower protein detection could limit its scalability for ambitious environmental projects."

Table 1. Add more results from Table 1 in the Results section such as the cost comparison?

Response:

We thank the reviewer for this suggestion. To address this concern, we have created a dedicated cost-efficiency figure (Figure 6) that provides a clear and straightforward visual comparison of all library preparation kits and PacBio HiFi sequencing across four biologically meaningful metrics: cost per Gb of trimmed sequencing data, cost per 1,000 unique genes, cost per high-quality MAG, and cost per total MAG. All methodological details regarding cost calculation are provided in the figure legend. This figure offers readers an immediate and intuitive interpretation of the cost data contained in Table 1, which was previously underutilized in the manuscript.

To support this figure, the following additions have been made to the manuscript:

A Cost-Efficiency Analysis subsection has been added to the Results section (lines 621–648 of the revised manuscript):

"To incorporate the economic dimension, we performed a concise cost-effectiveness analysis across four biological output metrics to compare library-preparation kits and sequencing platforms (Figure 6). When normalized to sequencing yield, TruSeq-100 at 250PE was the most cost-efficient kit ($63.4 per Gb trimmed), representing a 1.5-fold improvement over the least efficient kit (NEB-1 at 150PE; $96.0 per Gb). PacBio HiFi sequencing was the most expensive per Gb trimmed ($219.2), approximately 3.5-fold higher than TruSeq-250PE. The cost advantage of Illumina 250PE sequencing was most pronounced for unique gene discovery: TruSeq-250PE achieved a cost of $1.35 per 1,000 unique genes, compared to $27.37 for PacBio HiFi, a 20-fold difference, reflecting the substantially larger gene catalogue recovered by this kit. For high-quality MAG recovery, TruSeq-250PE ($99.8 per HQ MAG) and PacBio HiFi ($133.4 per HQ MAG) were broadly comparable, while NEB-1 performed poorly regardless of read length ($948 to $1,068 per HQ MAG). In contrast, when considering total MAG yield, long-read sequencing was markedly more cost-efficient ($7.35 per total MAG vs. $30.50 for TruSeq-250PE), owing to the 327 MAGs recovered by PacBio HiFi."

In addition, a brief interpretive paragraph has been added to the Discussion (lines 856–862 of the revised manuscript):

"Although third-generation LRS offers substantial advantages for genome assembly and MAG recovery, cost and sample requirements must also be considered. At the time our study was conducted, PacBio HiFi was approximately 2.2× more expensive per sample than TruSeq 2×250 bp, and while it provided unmatched cost-efficiency for total MAG yield, Illumina 250PE sequencing was markedly more cost-effective for gene discovery and high-quality genome recovery, making it the preferred strategy for large-scale metagenomic studies prioritising functional diversity."

I think most other tables could go to the supplement and instead have a figure with a couple panels in the main document that show the most important results of the tables (e.g., N50, number of contigs obtained) with bars/columns, which will make it easier to communicate the results.

Response:
We thank the reviewer for this constructive suggestion. We fully agree that moving detailed assembly metrics tables to the supplementary material and replacing them with a visual summary in the main text will substantially improve the readability and impact of the manuscript. We have implemented the following changes:

First, we have created a new multi-panel figure (Figure 2) that displays the most important assembly metrics (total assembly size in Mbp, number of contigs in $\times 10^3$, and N50 in bp) across all six Illumina-compatible library preparation methods at both read lengths (150PE and 250PE), alongside the global merge (GM) co-assemblies and PacBio HiFi long-read sequencing. This hybrid figure integrates per-kit replicate data with error bars alongside co-assembly reference

values, providing a comprehensive yet accessible visual summary of the assembly performance data previously reported across Tables 3, 4, 5, and 6.

Second, Tables 3, 4, 5, and 6 have been consolidated into a single Supplementary Table 1, which retains all original data for reference. The previous Supplementary Tables 1 and 2 have been renamed to Supplementary Tables 2 and 3, respectively, to accommodate this addition.

All in-text citations have been updated accordingly throughout the revised manuscript.

Reviewer #3:

Thank you for submitting this manuscript. The comparative assessment of library preparation kits and sequencing technologies is valuable, but several areas would benefit from clarification or refinement.

1. The description of the environmental sample as "unique and highly diverse" would benefit from simplification. "Diverse" is sufficient, as "highly" is unquantified and "unique" applies to any combined samples.

Response:

We thank the reviewer for this observation and agree that the original description was imprecise. We have revised all relevant instances throughout the manuscript accordingly. Specifically, "unique and highly diverse mock environmental sample was engineered" has been replaced with "diverse composite sample of mangrove sediment and palm tree soil was assembled" in the Methods section, and "engineered" has been replaced with "composite" in the Results section, where the sample is referenced again. Additionally, "highly diverse" has been simplified in the Discussion, in reference to the sample. The revised text now reads:

"A diverse composite sample of mangrove sediment and palm tree soil was assembled to provide sufficient DNA quantity and quality for comprehensive testing across various library preparation kits and sequencing technologies without being influenced by different sampling sites and DNA extraction." (line 171 in the revised manuscript)

"DNA extraction from the composite terrestrial soil and marine sediment mixture yielded a total of 6.2 µg DNA." (line 386 in the revised manuscript)

"The present study sought to maximize data acquisition and expand biological understanding in metagenomic analyses of complex and diverse environmental samples." (line 653 in the revised manuscript)

"Our key benchmarking finding indicates that the TruSeq Illumina library preparation kit, when combined with a 250PE sequencing read length, can yield results that closely align with those obtained using long-read technologies for high-quality MAGs in a diverse environmental sample." (line 865 in the revised manuscript)

2. Additional methodological detail is required regarding sample collection and handling. Please specify the collection sites, storage conditions, and whether samples were dried or otherwise processed or homogenised prior to DNA extraction.

Response:

We thank the reviewer for this request and have expanded the Methods section accordingly to include collection sites, coordinates, sample handling procedures, and DNA extraction details. The revised text now reads (lines 179–188 in the revised manuscript):

"Samples were collected on 25 June 2021 from two distinct environments located inside the King Abdullah University of Science and Technology (KAUST) campus (Thuwal, Saudi Arabia): marine mangrove sediments (22°19'19.7"N, 39°06'08.7"E) and terrestrial palm tree soil (22°18'26.7"N, 39°06'16.0"E). Samples were collected from the upper 2 cm of the soil/sediment surface using

sterilized spatulas, placed into sterilized 50 ml conical tubes, and processed immediately after collection without prior drying. A manual mixing step was performed prior to weighing and DNA extraction. For each environment, five independent extractions were carried out with the DNeasy PowerMax Soil Kit (Qiagen, Hilden, Germany), following the manufacturer's instructions and using 8 grams of mixed material per extraction."

3. Only one binning programme is used prior to dastool refinement. It would be helpful to explain this choice, particularly as relying on a single tool may omit bins that alternative programmes such as concoct or maxbin could recover and dastool could then combine. As is, it is possible that variation and differences are influenced by metabat2 clustering algorithms.

Response:

We thank the reviewer for this observation. MetaBAT2 was deliberately selected as the binning tool for all sequencing conditions based on two main considerations. First, at the time of our experiment and analysis, it was the most widely used binning tool compatible with both short-read and long-read data, enabling consistent and unbiased cross-technology benchmarking under identical conditions across all 13 sequencing conditions evaluated in this study. Second, MetaBAT2 has been recognized as one of the most computationally efficient binners across multiple data types and binning modes, making it well-suited for large-scale comparative studies (Kang et al. 2019; Han et al. 2025).

Following the reviewer's suggestion, MetaWRAP was run on all short-read datasets as a complementary multi-binner validation approach. MetaWRAP was not applied to the PacBio long-read dataset as it does not support long-read data. The short-read results are presented in Supplementary Table 1-c. As expected from the combination of three independent binners, MetaWRAP recovered a higher absolute number of MAGs than MetaBAT2 alone; however, the relative performance trends across kits and sequencing lengths were fully consistent with the MetaBAT2 results, demonstrating that the observed differences are driven by library preparation and sequencing parameters rather than by the choice of clustering algorithm.

The following sentences have been added to the Materials and Methods section (lines 320–331 in the revised manuscript):

"MetaBAT2 was selected for binning across all sequencing conditions as it was, at the time of analysis, the most widely used tool compatible with both short- and long-read data, enabling consistent cross-technology comparisons, and has been recognized as one of the most computationally efficient binners across multiple data types and binning modes (Kang et al. 2019; Han et al. 2025). To validate that the observed differences were not influenced by the choice of binning algorithm, MetaWRAP (which integrates MetaBAT2, MaxBin2, and CONCOCT) was additionally applied to all short-read datasets, with results presented in Supplementary Table 1-c. MetaWRAP was not applied to the PacBio long-read dataset as it does not support long-read data. Completeness and contamination were performed with CheckM v1.2.2 (Parks et al. 2015). For benchmarking comparison, high-quality MAGs were defined as bins with completeness >90%, contamination <5%."

4. Version numbers should be provided for all software tools.

Response:
We thank the reviewer for this observation. We have revised the Materials and Methods section to include version numbers for all software tools used in this study. The revised text appears at the corresponding lines of the revised manuscript.

5. Taxonomic names should be italicised consistently throughout the manuscript.

Response:
We thank the reviewer for this observation. We have systematically reviewed the entire manuscript and corrected all taxonomic names to italics throughout the text and figure legends. The corrections affect the following locations:

- Results, Novel MAGs section (lines 560–562 in the revised manuscript): *Planctomycetota*, *Actinobacteriota*, *Pirellulaceae*, *Vicingaceae*, *Bacteroidota*
- Supplementary Figure 3 legend (lines 1078–1081 in the revised manuscript): *Planctomycetota*, *Rubrobacter*, *Planctomycetaceae*, *Bacteroidales*

Note: GTDB placeholder identifiers (e.g., g__JABFRZ01, f__GCA-002686595, g__VAYN01, g__UBA11591) are database identifiers, not formal taxonomic names, and have therfore not been italicised.

6. The manuscript alternates between using the sample as a benchmarking resource and as a biological discovery dataset (e.g., the reporting of novel taxa and detection candidate gene families). The narrative would benefit from clearer distinction between these aims.

Response:
We thank the reviewer for this observation. We agree that the manuscript would benefit from a clearer narrative distinction between its two complementary aims: the methodological benchmarking of library preparation kits and sequencing strategies, and the biological discoveries enabled by optimal sequencing conditions. In response, we have made the following targeted revisions throughout the Results and Discussion to explicitly separate these two dimensions, clearly labeling which statements reflect technical benchmarking findings and which represent biological consequences or discoveries.

In the Results section, the following revisions have been made:

- The Taxonomy subsection has been expanded to include the GC content correlation analysis, explicitly distinguishing the mechanistic basis of Nextera differential abundance from its biological consequence. As detailed in our response to Reviewer #2, Comment on lines 317–350 of this document, the following sentence has been added immediately after the description of the differential abundance analysis (revised lines 464–468):

"Analysis of the genomic GC content of these differential abundance taxa revealed a significant positive correlation between GC content and fold change at 150PE (Spearman $\rho = +0.615$, $p = 0.033$), with over-represented genera showing a mean GC of 68.8% compared to 48.4% for under-represented genera, while no significant correlation was observed at 250PE ($\rho = -0.049$, $p = 0.880$)."

- The novel MAGs subsection has been revised to explicitly frame the novel species findings as biological discoveries enabled by the optimal sequencing strategy, rather than as benchmarking results (revised lines 555–559):

"These results suggested these MAGs could be potential novel species, a biological discovery made possible by the genome assembly quality achieved with long-read sequencing. We then set out to verify whether the same novel lineages were recoverable using the short-read strategies benchmarked in this study, thereby assessing the capacity of each sequencing condition to access this layer of biological diversity."

- The PacBio diversity statement has been revised to clarify that lower diversity is a technical sampling effect, not a biological finding (revised lines 481–483):

"As expected, given its lower total read count relative to the SRS datasets, the PacBio library exhibited the lowest detected genus-level diversity (Figure 3-C1), reflecting depth-dependent sampling rather than a biological difference in community composition."

- The protein detection statement has been revised to distinguish a known technical feature of long-read sequencing from a biological difference in community content (revised lines 523–527):

"This reflects a known characteristic of long-read sequencing, whereby organisms present at low abundance are less efficiently assembled and their genes less completely recovered at standard sequencing depths (Kim et al. 2024), rather than a biological difference in the functional content of the community."

In the Discussion, the following targeted revisions have been made:

- The opening summary paragraph has been revised to explicitly separate mechanistic cause from biological consequence (revised lines 666–673):

" We found that the results were considerably influenced by the fragment size distribution of the libraries generated by the kits, which determines the effective insert length available for sequencing. Mechanistically, fragment-size differences reflect the fragmentation chemistry used (physical shearing, enzymatic digestion, or transposase-mediated tagmentation), each imposing distinct, reproducible biases that affect assembly, binning, and annotation. Biologically, these biases manifest as altered adapter contamination, assembly quality, gene coverage, and MAG reconstruction."

- The fragmentation chemistry paragraph has been revised, replacing the original closing sentence (revised lines 727–734):

"On the other hand, and consistent with observations related to enzyme-based fragmentation technologies, Gunasekera et al. reported biases in coverage, GC content, and de novo assembly quality metrics also when using tagmentation methods (Gunasekera et al. 2021). Consistent with these findings, a significant GC-dependent bias was observed in the differential abundance analysis of Nextera libraries at 150PE but not at 250PE, a pattern that may reflect the improved

assembly of a broader range of genomic regions enabled by longer reads, which could reduce the relative impact of fragmentation-associated GC bias on taxonomic recovery."

- The *de novo* assembly paragraph final sentence has been revised to explicitly label the biological consequence of a mechanistic process (revised lines 770–774):

"Technically, library preparation-level sequencing bias propagates through the de Bruijn graph assembly, amplifying coverage unevenness into graph complexity that assembly algorithms cannot fully resolve. Biologically, this results in higher contamination rates and fewer high-quality MAGs in enzymatic fragmentation datasets, a pattern directly confirmed in our results."

- The concluding Discussion paragraph has been revised to separate benchmarking outcomes from biological discoveries (revised lines 863–867):

"Our key benchmarking finding indicates that the Illumina TruSeq library preparation kit, when combined with a 250PE sequencing read length, can yield results that closely align with those obtained using long-read technologies for high-quality MAGs in a diverse environmental sample, while recovering almost tenfold more unique proteins than PacBio HiFi."

7. Please clarify whether a hybrid assembly was considered, as this could potentially improve completeness metrics.

Response:

We thank the reviewer for this constructive suggestion. However, the scope of the present study was deliberately designed as a benchmarking comparison of Illumina-compatible library preparation methods and read lengths against PacBio HiFi long-read sequencing as a reference technology, rather than as a biological experiment aimed at maximizing assembly and binning completeness. In this context, hybrid assembly was outside the intended experimental framework, as its inclusion would have introduced an additional variable that could confound the direct comparison between short-read and long-read strategies evaluated here.

8. Several conclusions would be strengthened by more mechanistic interpretation, particularly around why specific kits perform better or worse beyond fragment size distribution.

Response:

We thank the reviewer for this important and constructive observation. We agree that the original manuscript did not sufficiently explain why specific kits perform better or worse beyond fragment size distribution. In response, we have made the following targeted substitutions in the Results and Discussion, grounded in four mechanistic factors: fragmentation chemistry, size selection, PCR duplication rates, and GC-dependent bias. Several of these revisions are shared with the dual narrative comment above and are cross-referenced accordingly.

In the Results, the following revisions have been made:

- The Library Preparation subsection has been revised to replace tentative phrasing with a direct mechanistic statement (revised lines 394–396):

"This mechanistically accounts for the higher PCR duplication rates and library yields observed in the Ovation and Nextera datasets."

- A new sentence has been added at the end of the sequencing outcomes paragraph to report the newly identified finding on PCR duplication rates (revised lines 407–409):

"Notably, a consistent and statistically significant decrease in PCR duplicate rates was observed across all kits at 250PE compared to 150PE, suggesting that longer read lengths improve library complexity independently of kit chemistry."

In the Discussion, the following sentence substitutions have been made:

- The opening Discussion paragraph: *"largely influenced"* has been replaced with *"considerably influenced"* (revised line 667).
- The fragmentation chemistry paragraph final sentence and the de novo assembly paragraph final sentence have been revised as detailed in our response to the **Dual Narrative comment above**, where both sentences were replaced with explicit mechanistic and biological interpretations.
- The TruSeq performance paragraph: the opening and final sentences have been replaced:

Revised opening (lines 775–776 in the revised manuscript): *"The superior performance of the TruSeq kit can be attributed to a combination of mechanistic factors beyond fragment size distribution."*

Revised final sentence (lines 782–786 in the revised manuscript): *"TruSeq consistently exhibited the lowest PCR duplication rates across both read lengths, even without reducing PCR cycles, and its size-selection step (absent in the Ovation protocol despite equivalent physical shearing) independently improves flow-cell clustering efficiency, confirming that fragment size alone does not account for the performance differences observed."*

9. High quality MAGs should include full rRNA operons and sufficient tRNA representation. Please confirm whether these criteria were assessed alongside CheckM statistics.

Response:

We thank the reviewer for this suggestion. We have now performed rRNA and tRNA gene annotation for all four representative MAG pairs recovered from both KM-TruSeq-250PE and PacBio HiFi assemblies using barrnap (v1.10.5; https://github.com/tseemann/barrnap). The results are summarized in the newly added Supplementary Table 1-d. The following sentences have been added to the MAG Reconstruction, Classification, and Comparison subsection of the Materials and Methods (lines 344–349 in the revised manuscript):

"To further consolidate the high quality of these selected MAGs, rRNA and tRNA gene annotation of the selected high-quality MAGs was performed using barrnap (v1.10.5; https://github.com/tseemann/barrnap). Results are summarized in Supplementary Table 1-d. In line with MIMAG standards (Bowers et al. 2017), high-quality MAGs were additionally required to contain rRNA genes and a minimum of 18 tRNA genes."

As expected, PacBio HiFi assemblies recovered higher rRNA and tRNA gene counts than their KM-TruSeq-250PE counterparts across all four MAG pairs (Supplementary Table 1-d), further consolidating the superiority of long-read sequencing for high-quality MAG reconstruction.

10. The text could be made more concise. There are several sections where results are repeated across the main text, tables, and figures, and streamlining could possibly improve clarity.

Response:

We thank the reviewer for this constructive suggestion. We have carefully reviewed the manuscript and identified sections where results were redundantly described across the main text, tables, and figures. Given that several tables have now been consolidated into Supplementary Table 1 and two new figures (Figure 2, assembly metrics; Figure 6, cost-efficiency) have been added, a number of originally descriptive passages have become redundant. To avoid modifying text that was already revised in response to other comments, we focused our conciseness edits exclusively on original text where duplication or over-explanation was most evident.

In the Introduction, the following revisions have been made:

- The following sentence has been removed as it is redundant with the preceding sentence describing the scope of metagenomics:

"Metagenomics offers a snapshot of the true genetic diversity within an ecosystem (Stein et al. 1996) and captures microbial life in its natural habitats, from highly diverse microbial populations in soil (Daniel 2005) to the intricacies of the human gut microbiome (Almeida et al. 2021)."

- The following two sentences have been removed as they repeat points already made in the preceding paragraph about the limitations of mock communities, with the Sanders et al. 2019 citation retained in the Methods section:

"Sanders et al. emphasized that authentic environmental samples provide a more realistic framework for evaluating library preparation methods and sequencing technologies. They used long-read sequencing as a benchmarking in environments permitting a realistic assessment of sequencing and assembly strategies for novel and complex microbial communities (Sanders et al. 2019)."

- The following sentence has been removed from the closing paragraph of the Introduction as it repeats the aim already stated in the preceding sentence:

"By integrating these results, we aimed to identify sequencing strategies that maximize the recovery of taxonomic, functional, and genome-resolved microbial information from complex metagenomes."

In the Methods, the following revisions have been made:

- The OrthoVenn3 description has been condensed by removing two redundant sentences (lines 350–352 in the revised manuscript):

"Orthologous gene clusters among the selected bins were identified using OrthoVenn3 (Sun et al. 2023) with default parameters."

In the Results, the following revisions have been made:

- In the subsection *"Metagenome-Assembled Genomes"*, we streamlined the opening paragraph, which contained granular kit-by-kit MAG counts and quality percentages that are visually conveyed by Figure 5-A. We retained all numerically specific statements that are not represented in figures, as well as all text added during this revision (lines 539–545 in the revised manuscript):

"We were also interested in measuring the effect of SRS versus LRS on MAG recovery. As shown in Figure 5-A, LRS produced substantially more MAGs than either GM or KM assemblies despite lower sequencing depth, because longer reads span repetitive regions and reduce graph complexity in de novo assembly algorithms, resulting in more contiguous and accurate genome reconstructions. Shorter library inserts consistently yielded fewer and lower-quality MAGs across SRS kits, with MAG absolute recovery and quality highest in the TruSeq-250PE and Nextera-250PE KM combinations."

- The following sentence has been removed from the Novel MAGs subsection as it is redundant with the preceding sentence, which was updated to include the word "underexplored" (line 598 in the revised manuscript):

"Their detection highlights the presence of underexplored microbial diversity in the sample."

In the Discussion, the following revisions have been made:

- The following two sentences have been removed from the fragmentation chemistry paragraph as they duplicate information already covered by Tanaka et al. 2020, cited in the preceding sentence:

"A previous report indicated that a fragmentase (developed by New England Biolabs) generates more artifactual indels than sonication or nebulization. The number of indels produced by fragmentase was within a two-fold range of those generated by the physical methods (Knierim et al. 2011)."

- The following two sentences have been removed from the TruSeq performance paragraph as they are too general and superseded by the mechanistic interpretation response added above:

"The library prep protocols have many phases, largely divided in end-repair, A-tailing, ligation, index addition, and final amplification. At any of those, the difference in reagents among the manufacturers may have affected the quality of the final libraries."

In the Conclusion, the following revisions have been made:

- The following four sentences have been removed as they repeat Nextera-specific information already covered more concisely in the kit recommendations paragraph added in response to Reviewer #2:

"In our opinion, the Nextera kit is also a suitable option, particularly for low-input samples, showing competitive performance in both protein and MAG recovery. However, the taxonomic

discrepancies observed in Nextera-prepared samples could raise concerns that warrant further investigation. Additionally, the streamlined nature of the Nextera workflow makes it well-suited for automation. Nevertheless, it is important to note that tagmentation-based technologies can be susceptible to the presence of inhibitors, particularly present in environmental samples, which may negatively impact the library preparation process."

Re: Spectrum00013-26R1 (**COMPARATIVE METAGENOMIC ASSESSMENT OF ILLUMINA-COMPATIBLE LIBRARY PREPARATION METHODS, SHORT-READ LENGTHS, AND PACBIO HIFI REVEALS UNKNOWN MICROBIAL INFORMATION IN A COMPLEX ENVIRONMENTAL SAMPLE**)

Dear Dr. Rubén Díaz-Rúa:

Thank you for the privilege of reviewing your work. Below you will find my comments, instructions from the Spectrum editorial office, and the reviewer comments.

The revisions have substantially strengthened the manuscript however, please address the remaining reviewer comments.

Please return the manuscript within 60 days; if you cannot complete the modification within this time period, please contact me. If you do not wish to modify the manuscript and prefer to submit it to another journal, notify me immediately so that the manuscript may be formally withdrawn from consideration by Spectrum.

Revision Guidelines
To submit your modified manuscript, log into the submission site at https://spectrum.msubmit.net/cgi-bin/main.plex. Go to Author Tasks and click the appropriate manuscript title to begin. The information you entered when you first submitted the paper will be displayed; update this as necessary. Note the following requirements:

• Upload point-by-point responses to the issues raised by the reviewers in a file named "Response to Reviewers," NOT in your cover letter.
• Upload a compare copy of the manuscript (without figures) as a "Marked-Up Manuscript" file.
• Upload a clean .DOC/.DOCX version of the revised manuscript and remove the previous version.
• Each figure must be uploaded as a separate, editable, high-resolution file (TIFF or EPS preferred), and any multipanel figures must be assembled into one file.
• Any supplemental material intended for posting by ASM should be uploaded with their legends separate from the main manuscript. You can combine all supplemental material into one file (preferred) or split it into a maximum of 10 files with all associated legends included.

For complete guidelines on revision requirements, see our Submission and Review Process webpage. Submission of a paper that does not conform to guidelines may delay acceptance of your manuscript.

Data availability: ASM policy requires that data be available to the public upon online posting of the article, so please verify all links to sequence records, if present, and make sure that each number retrieves the full record of the data. If a new accession number is not linked or a link is broken, provide Spectrum production staff with the correct URL for the record. If the accession numbers for new data are not publicly accessible before the expected online posting of the article, publication may be delayed; please contact production staff (Spectrum@asmusa.org) immediately with the expected release date.

Publication Fees: For information on publication fees and which article types are subject to charges, visit our website. If your manuscript is accepted for publication and any fees apply, you will be contacted separately about payment during the production process; please follow the instructions in that e-mail. Arrangements for payment must be made before your article is published.

ASM Membership: Corresponding authors may join or renew ASM membership to obtain discounts on publication fees. Need to upgrade your membership level? Please contact Customer Service at Service@asmusa.org.

The ASM Journals program strives for constant improvement in our submission and publication process. Please tell us how we can improve your experience by taking this quick Author Survey.

Thank you for submitting your paper to Spectrum.

Sincerely,
Vanessa Varaljay
Editor
Microbiology Spectrum

Reviewer #2 (Comments for the Author):

The revised article by Díaz-Rúa represents a significantly improved version of the originally submission, and the authors have addressed all my major and minor concerns. I also want to specially thank the authors for adding more results and discussion to address my major concerns such as providing the cost assessment for each approach/protocol, details about how the composite

sample was put together, and the GC% of the differentially abundant taxa between the protocols. I believe that these edits added value to the study. I do not have any remaining major concerns regarding the manuscript or the revisions performed. I have only a few, mostly esthetic comments, for the authors to consider at their discretion.

Title: I appreciate the authors' effort to improve the title of the manuscript, and I think they have somewhat succeed in this, but the last part of the title remains a bit vague to me. What unknown information, and unknown compared to what? How about to replace that part with "Comparisons....on the same environment DNA sample" or "reveals different diversity recovered by each method?" or something like this?
Line 23. "with Illumina-compatible libraries prepared in triplicate." I think this part is not really needed; add "triplicate" in the first part of the sentence, I suggest.
Line 31. What BGC stands for?
Line 46. long-read PacBio sequencing. (add "PacBio" to be more specific).

Reviewer #3 (Comments for the Author):

The manuscript has improved following revisions and I thank the authors for considering the proposed edits. There are some remaining edits that I think would either help with the clarity of the science of the manuscript and make the science presented more robust.

Proposed changes:
The introduction still frames metagenomics as a field when it is a technique and as recent advance when it has now been around for a reasonable time frame - perhaps it would be useful to frame metagenomics in this manner and less as a new emerging area of research. In this context the lack of benchmarking is more of an issue that the paper is working to resolve.

104 a couple of bracketed examples of matrices might be useful to illustrate the point about how matrices can vary between environments

344 the cost efficiency is possibly very dependant on extraneous factors and timing - it would be worth adding a statement to make it at explicit that these are the costs at time of writing, because whilst this is a relevant thing to compare some labs will sequence in-house and others will outsource.

431 it would be beneficial to also run AAI analysis or to use the TYGS server in order to assess the species relatedness in cases where taxonomic novelty is being proposed

There is definite merging of results and discussion at several points in the manuscript - it may be beneficial to ensure these sections are separated or to merge so there is greater coherence.

Minor

References occasionally use first initial

Terms like notably and interestingly are subjective and should ideally be avoided if possible.

Re: Spectrum00013-26R1 (**COMPARATIVE METAGENOMIC ASSESSMENT OF ILLUMINA-COMPATIBLE LIBRARY PREPARATION METHODS, SHORT-READ LENGTHS, AND PACBIO HIFI REVEALS UNKNOWN MICROBIAL INFORMATION IN A COMPLEX ENVIRONMENTAL SAMPLE**)

Editor Comments:

Thank you for the privilege of reviewing your work. Below you will find my comments, instructions from the Spectrum editorial office, and the reviewer comments.
The revisions have substantially strengthened the manuscript however, please address the remaining reviewer comments.
Please return the manuscript within 60 days; if you cannot complete the modification within this time period, please contact me. If you do not wish to modify the manuscript and prefer to submit it to another journal, notify me immediately so that the manuscript may be formally withdrawn from consideration by Spectrum.

General response to the editor and all reviewers:

We would like to thank the editor and reviewers for their continued and constructive engagement with our manuscript. Their remaining comments have helped us further strengthen the clarity, scientific rigor, and presentation of our study.

In the following section, we provide a detailed point-by-point response to each comment raised by the reviewers.

Reviewer #2:

The revised article by Díaz-Rúa represents a significantly improved version of the originally submission, and the authors have addressed all my major and minor concerns. I also want to specially thank the authors for adding more results and discussion to address my major concerns such as providing the cost assessment for each approach/protocol, details about how the composite sample was put together, and the GC% of the differentially abundant taxa between the protocols. I believe that these edits added value to the study. I do not have any remaining major concerns regarding the manuscript or the revisions performed. I have only a few, mostly esthetic comments, for the authors to consider at their discretion.

Title: I appreciate the authors' effort to improve the title of the manuscript, and I think they have somewhat succeed in this, but the last part of the title remains a bit vague to me. What unknown information, and unknown compared to what? How about to replace that part with "Comparisons....on the same environment DNA

Response:

We thank the reviewer for this suggestion. "We have revised the last part of the title from "reveals unknown microbial information" to "reveals differences in microbial and functional diversity recovery", which more precisely conveys that both the taxonomic and functional dimensions of the microbial community are differentially captured depending on the methodological choices made. The new title reads:

"*Comparative metagenomic assessment of Illumina-compatible library preparation methods, short-read lengths, and PacBio HiFi sequencing reveals differences in microbial and functional diversity recovery from a complex environmental sample*"

Line 23. "with Illumina-compatible libraries prepared in triplicate." I think this part is not really needed; add "triplicate" in the first part of the sentence, I suggest.

Response:

We thank the reviewer, confirm the relocation of "in triplicate", and show the revised sentence (lines 26-27 of the revised manuscript):

"Here, we benchmarked six Illumina-compatible short-read library preparation conditions in triplicate at 2×150 bp and 2×250 bp read lengths alongside PacBio HiFi long-read sequencing using a composite environmental sample of marine mangrove sediment and terrestrial palm tree soil."

Line 31. What BGC stands for?

Response:

We thank the reviewer for this observation. We have defined the acronym on first use, now reading: "biosynthetic gene cluster (BGC)". Line 37 of the revised manuscript:

"Furthermore, biosynthetic gene cluster (BGC) analysis identified 46 BGCs in TruSeq-250PE assemblies compared to 38 in PacBio HiFi, with several showing no close match in the MIBiG database."

Line 46. long-read PacBio sequencing. (add "PacBio" to be more specific).

Response:

We thank the reviewer for this suggestion. We have added "PacBio HiFi" before "long-read sequencing" for specificity. Line 51 of the revised manuscript.

<u>Reviewer #3:</u>

The manuscript has improved following revisions and I thank the authors for considering the proposed edits. There are some remaining edits that I think would either help with the clarity of the science of the manuscript and make the science presented more robust.

The introduction still frames metagenomics as a field when it is a technique and as recent advance when it has now been around for a reasonable time frame - perhaps it would be useful to frame metagenomics in this manner and less as a new emerging area of research. In this context the lack of benchmarking is more of an issue that the paper is working to resolve.

Response:

We thank the reviewer for this observation. We have revised the Introduction to better reflect the established nature of metagenomics, replacing "novel scientific field" with "scientific approach". The revised sentence now reads:

"The application of high-throughput sequencing for studying microbial communities has established metagenomics as a scientific approach for analyzing the collective genomes of organisms coexisting within a given environment." (lines 68 and 69 in the revised manuscript)

104 a couple of bracketed examples of matrices might be useful to illustrate the point about how matrices can vary between environments

Response:

We thank the reviewer for this suggestion. We have expanded the sentence to include both examples of common environmental matrices and their associated chemical inhibitors. In addition, we have added supporting references covering soil (Galla et al. 2024), coral (Weber et al. 2017), biofilm (Florczyk et al. 2022), and matrix-dependent library preparation challenges (Gaulke et al. 2021). The revised sentence now reads:

" In addition, the matrix in which microbial communities are often embedded (e.g., soil, coral, or biofilm), and its associated inhibitors, such as humic acids, polysaccharides, and calcium ions, among others, can add additional challenges to successful sample processing and reproducibility (Gaulke et al. 2021; Galla et al. 2024; Weber et al. 2017; Florczyk et al. 2022*)." (*lines 111-114 in the revised manuscript)

344 the cost efficiency is possibly very dependant on extraneous factors and timing - it would be worth adding a statement to make it at explicit that these are the costs at time of writing, because whilst this is a relevant thing to compare some labs will sequence in-house and others will outsource.

Response:

We thank the reviewer for this important observation. We acknowledge that this caveat was already present in the Figure 6 legend, though perhaps not sufficiently prominent. We have now added a dedicated sentence to the Library Preparation and Sequencing section of the Materials and Methods, which covers all cost figures reported throughout the manuscript:

*"All reagents and sequencing costs reported in this study reflect prices at the KAUST Bioscience Core Lab (BCL) at the time the experiments were performed and may vary across institutions and over time." (*lines 179-181 in the revised manuscript)

431 it would be beneficial to also run AAI analysis or to use the TYGS server in order to assess the species relatedness in cases where taxonomic novelty is being proposed

Response:

We thank the reviewer for this suggestion, which certainly will strengthen our novelty claim. In addition to the ANI values already reported, we submitted the four high-quality PacBio LRS MAGs to the Type (Strain) Genome Server (TYGS; Meier-Kolthoff and Göker 2019; Meier-Kolthoff et al. 2022; Freese et al. 2025) for genome-based species delineation using digital DNA-DNA hybridization (dDDH). All four MAGs were classified as potential new species, with dDDH values (formula d4) well below the 70% species delineation threshold:

- MAG1 (g__JABFRZ01, Planctomycetota) — closest type strain identified by TYGS: Caldalkalibacillus thermarum (Firmicutes); dDDH 25.0%
- MAG2 (g__VAYN01, Actinobacteriota) — closest type strain identified by TYGS: Caldalkalibacillus thermarum (Firmicutes); dDDH 26.8%
- MAG3 (unclassified Pirellulaceae, Planctomycetota) — closest type strain identified by TYGS: Candidatus Laterigemmans baculatus (Planctomycetota); dDDH 25.4%
- MAG4 (g__UBA11591, Bacteroidota) — closest type strain identified by TYGS: Candidatus Sulfidibacterium hydrothermale (Bacteroidota); dDDH 31.1%

The cross-phylum assignment of the closest type strain for MAG1 and MAG2 reflects the extreme phylogenetic divergence of these MAGs from all currently characterized lineages, further underscoring their novelty. These results are consistent with the ANI and GTDB-Tk analyses already presented and collectively support the classification of these MAGs as genomically novel bacterial lineages.

The following section has been added to the Materials and Methods:

"In addition, the genome sequences were submitted to the Type (Strain) Genome Server (TYGS; https://tygs.dsmz.de) for whole genome-based species delineation (Meier-Kolthoff and Göker 2019; Meier-Kolthoff et al. 2022; Freese et al. 2025), using digital DNA-DNA hybridization (dDDH) formula d4 and species and subspecies delineation thresholds of 70% and 79%, respectively." Lines 279-283 of revised manuscript.

The following paragraph has been added to the Results, after the phylogenetic analysis of the four novel MAGs (lines 459-489 of the revised manuscript):

"*To further support the novelty of these four MAGs, we performed genome-based species delineation using TYGS. All four MAGs were classified as potential new species, with dDDH values (formula d4) against their closest type strains well below the 70% species delineation threshold: g__JABFRZ01 (closest type strain, Caldalkalibacillus thermarum, Firmicutes, dDDH 25.0%), g__VAYN01 (closest type strain, Caldalkalibacillus thermarum, Firmicutes, dDDH 26.8%), unclassified Pirellulaceae (closest type strain, Candidatus Laterigemmans baculatus, Planctomycetota, dDDH 25.4%), and g__UBA11591 (closest type strain, Candidatus Sulfidibacterium hydrothermale, Bacteroidota, dDDH 31.1%). The cross-phylum assignment of the closest type strain for g__JABFRZ01 and g__VAYN01 reflects the extreme phylogenetic divergence of these MAGs from all currently characterized lineages, further underscoring their novelty. These results are consistent with the ANI and phylogenetic analyses described above, collectively confirming that these MAGs represent genomically novel bacterial lineages.*"

There is definite merging of results and discussion at several points in the manuscript - it may be beneficial to ensure these sections are separated or to merge so there is greater coherence.

Response:

We thank the reviewer for this observation. We have carefully reviewed both sections and removed interpretive and mechanistic statements from the Results, either deleting them where they were already covered in the Discussion or moving them where they were not previously addressed. Specifically:

- The mechanistic explanation for LRS MAG superiority ("because longer reads span repetitive regions and reduce graph complexity in de novo assembly algorithms") has been removed from the Results, as this is already discussed in the context of de Bruijn graph assembly in the Discussion.
- The interpretive statement "a biological discovery made possible by the genome assembly quality achieved with long-read sequencing" has been removed from the Results, as it is already addressed in the Discussion.
- The classification of the four novel MAGs as microbial dark matter and the suggestion of novel natural product biosynthesis potential have been moved from the Results to the Discussion, where they are now presented as two new concluding sentences in the LRS/MAG paragraph. The updated paragraph now reads:

"*This strategy could provide a realistic framework for evaluating sequencing and assembly performance in complex and novel microbial communities, and for guiding future efforts aimed at maximizing overall MAG recovery. The recovery of four MAGs classified exclusively at high taxonomic ranks, with no cultured representatives, further highlights the potential of long-read sequencing to access microbial dark matter, lineages that remain largely uncharacterized and inaccessible to short-read approaches alone (Pavlopoulos et al. 2023). Furthermore, functional*

analysis revealed several biosynthetic gene clusters with no close match in the MIBiG database, suggesting the potential for novel natural product biosynthesis in these underexplored lineages." (Lines 669-677 of revised manuscript.)

- The concluding statement "these results further reinforce the status of long-read sequencing as the gold standard method for the recovery of high-quality MAGs" has been removed from the Results as it is already covered in the Discussion.
- The mechanistic explanation for lower LRS protein recovery has been removed from the Results. The corresponding Discussion paragraph has been strengthened with a bridging sentence that more clearly reconciles the apparent contradiction between LRS protein completeness and its known limitation with low-abundance organisms. The updated paragraph now reads:

"Despite this, the completeness of the proteins detected by the NGS-based kit was only 40%, whereas LRS reached 80%. However, this higher completeness must be interpreted in context, reflecting the quality of assembled genes rather than community-wide coverage. It is well-known that PacBio sequencing technology struggles with the recovery and assembly of organisms present at low abundance (C. Kim et al. 2024), meaning that while LRS produces more complete gene models for the organisms it assembles, it may miss a substantial fraction of the community entirely." (Lines 655-660 of revised manuscript.)

References occasionally use first initial

Response:
We thank the reviewer for this observation. The previous version of the manuscript used author-date citation style for readability during the review process, with first initials included where necessary to disambiguate authors sharing the same surname (e.g., multiple Kim, Kang, Li, and Robinson citations). The manuscript has now been reformatted to the ASM numbered citation style, which resolves all first initial inconsistencies.

Terms like notably and interestingly are subjective and should ideally be avoided if possible.

Response:
We thank the reviewer for this observation. We have removed all instances of subjective adverbs throughout the manuscript. Specifically, "Notably" has been removed from four instances in the Abstract and Results sections, and "Surprisingly" has been removed from one instance in the Results section. In each case the sentences have been restructured to stand independently without the adverb.

Re: Spectrum00013-26R2 (**COMPARATIVE METAGENOMIC ASSESSMENT OF ILLUMINA-COMPATIBLE LIBRARY PREPARATION METHODS, SHORT-READ LENGTHS, AND PACBIO HIFI SEQUENCING REVEALS DIFFERENCES IN MICROBIAL AND FUNCTIONAL DIVERSITY RECOVERY FROM A COMPLEX ENVIRONMENTAL SAMPLE**)

Dear Dr. Rubén Díaz-Rúa:

Thank you for submitting the revised version of your manuscript.

Your manuscript has been accepted, and I am forwarding it to the ASM production staff for publication. Your paper will first be checked to make sure all elements meet the technical requirements. ASM staff will contact you if anything needs to be revised before copyediting and production can begin. Otherwise, you will be notified when your proofs are ready to be viewed.

Data Availability: ASM policy requires that data be available to the public upon online posting of the article, so please verify all links to sequence records, if present, and make sure that each number retrieves the full record of the data. If a new accession number is not linked or a link is broken, provide production staff with the correct URL for the record. If the accession numbers for new data are not publicly accessible before the expected online posting of the article, publication may be delayed; please contact ASM production staff immediately with the expected release date.

Publication Fees: For information on publication fees and which article types have charges, please visit our website. We have partnered with Copyright Clearance Center (CCC) to collect author charges. If fees apply to your paper, you will receive a message from no-reply@copyright.com with further instructions. For questions related to paying charges through RightsLink, please contact CCC at ASM_Support@copyright.com or toll free at +1-877-622-5543. CCC makes every attempt to respond to all emails within 24 hours.

ASM Membership: Corresponding authors may join or renew ASM membership to obtain discounts on publication fees. Need to upgrade your membership level? Please contact Customer Service at Service@asmusa.org.

Embargo Policy: A press release may be issued as soon as the manuscript is posted on the Spectrum Latest Articles webpage. The corresponding author will receive an email with the subject line "ASM Journals Author Services Notification" when the article is available online.

The ASM Journals program strives for constant improvement in our submission and publication process. Please tell us how we can improve your experience by taking this quick Author Survey.

Thank you for submitting your paper to Spectrum.

Sincerely,
Vanessa Varaljay
Editor
Microbiology Spectrum
