# Supplementary material for: Comparative metagenomic assessment of Illumina-compatible library preparation methods, short-read lengths, and PacBio HiFi sequencing reveals differences in microbial and functional diversity recovery from a complex environmental sample
Source: Microbiol Spectr. 2026 Jul 27;14(9):e00013-26. doi: 10.1128/spectrum.00013-26 (PMC13532183; doi:10.1128/spectrum.00013-26)

Supplementary Figure 2

A) 150PE sequencing length

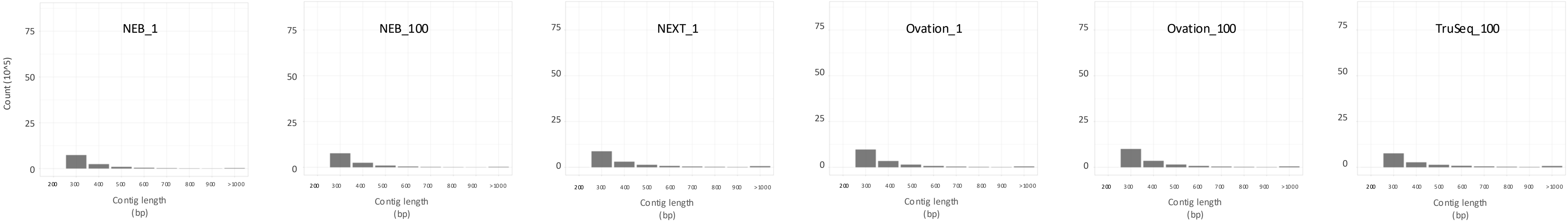

B) 250PE sequencing length

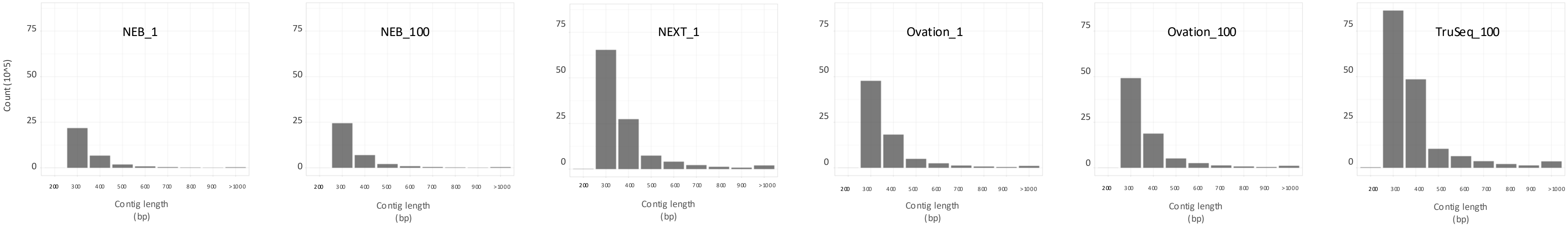

C) PacBio

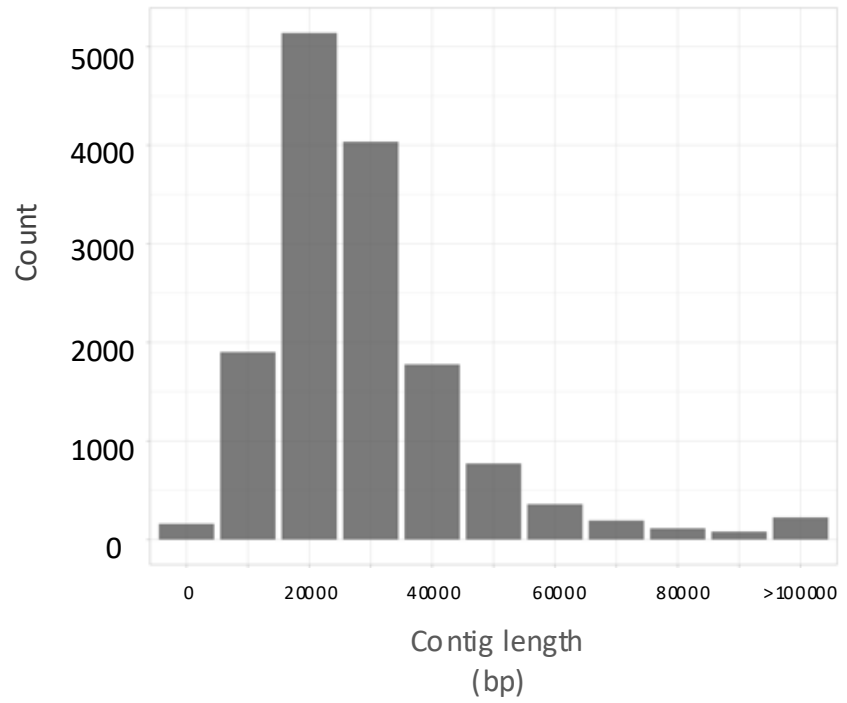

Supplement: Supplementary file 2 [file spectrum.00013-26-s0002.pdf]
