# Supplementary material for: Comparative metagenomic assessment of Illumina-compatible library preparation methods, short-read lengths, and PacBio HiFi sequencing reveals differences in microbial and functional diversity recovery from a complex environmental sample
Source: Microbiol Spectr. 2026 Jul 27;14(9):e00013-26. doi: 10.1128/spectrum.00013-26 (PMC13532183; doi:10.1128/spectrum.00013-26)

Supplementary Figure 3

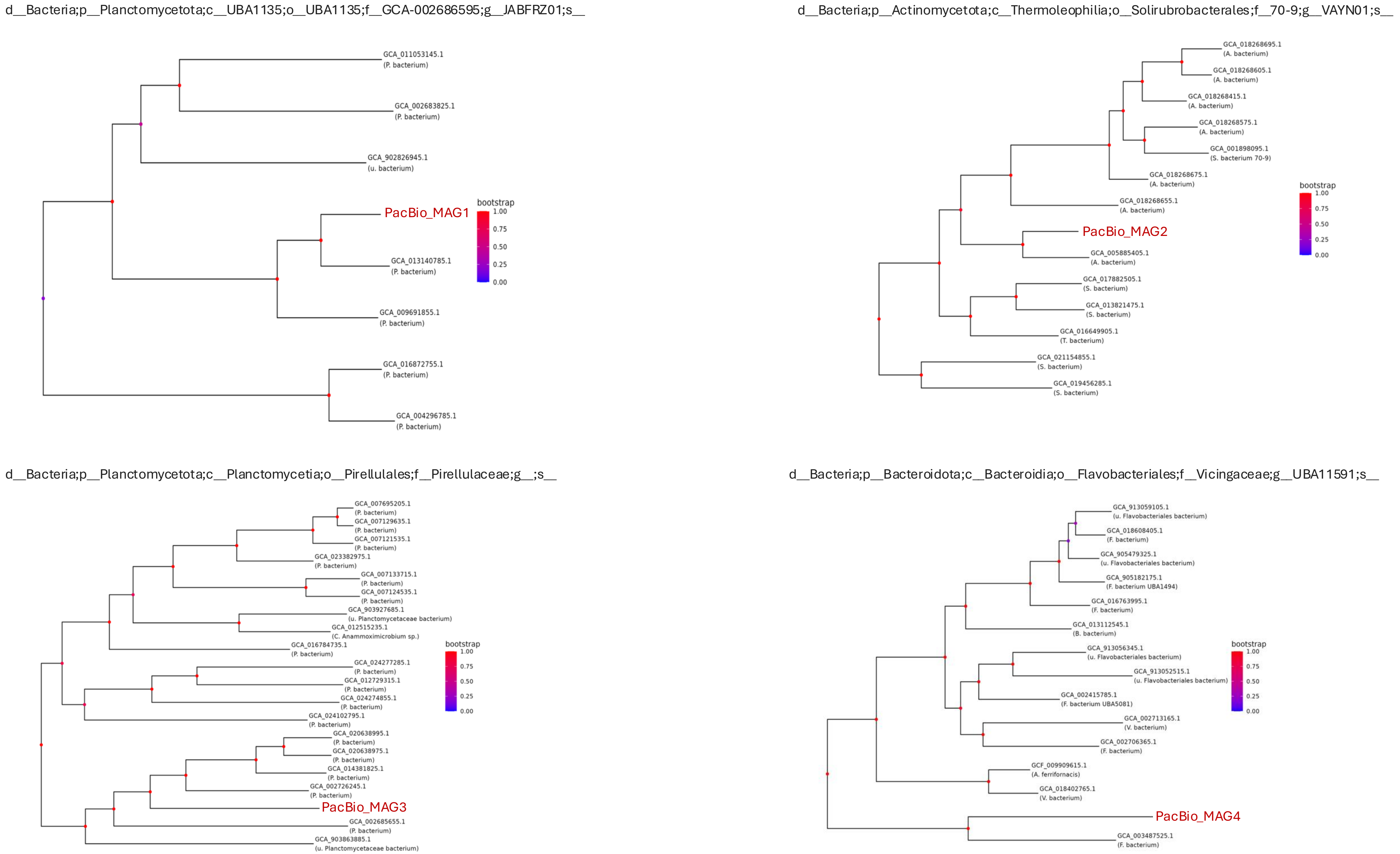

Supplement: Supplementary file 3 [file spectrum.00013-26-s0003.pdf]
