# Supplementary material for: Comparative metagenomic assessment of Illumina-compatible library preparation methods, short-read lengths, and PacBio HiFi sequencing reveals differences in microbial and functional diversity recovery from a complex environmental sample
Source: Microbiol Spectr. 2026 Jul 27;14(9):e00013-26. doi: 10.1128/spectrum.00013-26 (PMC13532183; doi:10.1128/spectrum.00013-26)
Supplement: Supplementary file 4 [file spectrum.00013-26-s0004.docx]

**SUPPLEMENTARY MATERIAL**

**Supplementary Figure 1.** Library preparation results. A) Bioanalyzer profiles showing the size distribution for each Illumina library preparation method. B) Final library concentration and number of PCR cycles used for each kit, following the manufacturer’s protocol. Library concentration was measured from 1 µL of the final library using the Qubit HS dsDNA assay. Bold numbers indicate the enrichment PCR cycles used for each Illumina SRS kit. C) Average library fragment size for each kit, calculated using the Bioanalyzer analysis software.

**Supplementary Figure 2.** Contig length distribution for the Illumina KM co-assemblies (A, 150PE; B, 250PE) and the PacBio assembly (C). Histograms of contig lengths were generated to assess and visualize assembly quality. The x-axis shows contig length (bp), and the y-axis shows the frequency (total number of contigs) within each length bin.

**Supplementary Figure 3.** Closest taxonomy assignment and phylogenetic placement for the four new MAGs from the PacBio dataset. For each genome, an appropriate outgroup was selected for phylogenetic analysis: PacBio_MAG1, p_*Planctomycetota*; PacBio_MAG2, g_*Rubrobacter*; PacBio_MAG3, f_*Planctomycetaceae*; PacBio_MAG4, o_*Bacteroidales*.

**Supplementary Table 2.** Comparison of MAG metrics between Illumina SRS KMs and PacBio HiFi LRS assemblies. Reported values include ANI (%), MASH distance (p-value), MAG quality, completeness (%) and contamination (%), N50 (Mb), total assembled genome size (kb), L50, number of contigs, number of predicted genes, and mean contig coverage.

**Supplementary Table 3.** Comparative analysis of biosynthetic gene clusters (BGCs) predicted by antiSMASH 8 in MAGs recovered from Illumina SRS KM-Truseq-250PE and PacBio HiFi LRS bins. The table summarizes the number and classes of BGCs identified (e.g., **NRPS, PKS, RiPP-like, terpene**, and other specialized metabolites). Predicted clusters are annotated based on similarity to known BGCs in the MIBiG database; percent similarity indicates the degree of homology.
