# Supplementary material for: Comparative metagenomic assessment of Illumina-compatible library preparation methods, short-read lengths, and PacBio HiFi sequencing reveals differences in microbial and functional diversity recovery from a complex environmental sample
Source: Microbiol Spectr. 2026 Jul 27;14(9):e00013-26. doi: 10.1128/spectrum.00013-26 (PMC13532183; doi:10.1128/spectrum.00013-26)
Supplement: Supplementary file 5 [file spectrum.00013-26-s0005.docx]

|  | **NEB_1** | **NEB_100** | **NEXT_1** | **Ovation_1** | **Ovation_100** | **TruSeq_100** | **ANOVA** |
| --- | --- | --- | --- | --- | --- | --- | --- |
| **Sum of assembly (Mbp)** |  |  |  |  |  |  |  |
| 150PE | 93 ± 2a | 111 ± 13b | 141 ± 3c | 139 ± 2c | 144 ± 5c | 129 ± 4b,c | L |
| 250PE | 438.5 ± 3.5a# | 472 ± 71a# | 1637 ± 52b# | 1057 ± 6c# | 1100 ± 50c# | 2605 ± 34d# | L |
| **Contigs (10^5)** |  |  |  |  |  |  |  |
| 150PE | 2.4 ± 0.05a | 2.8 ± 0.3b | 3.3 ± 0.1c | 3.4 ± 0.05c | 3.5 ± 0.1c | 2.9 ± 0.1c,b | L |
| 250PE | 11.6 ± 0.1a# | 12.5 ± 1.8a# | 40.7 ± 1.1b# | 27.0 ± 0.2c# | 28.0 ± 1.2c# | 62.6 ± 1.3d# | L |
| **Average (bp)** |  |  |  |  |  |  |  |
| 150PE | 392 ± 0.2a | 394 ± 3.3a | 427 ± 1.1b | 409 ± 0.1c | 410 ± 0.8c | 451 ± 1.4d | L |
| 250PE | 377.2 ± 0.3a# | 376 ± 1.5a# | 402 ± 1.7b# | 392 ± 0.1c# | 393 ± 0.5c# | 416 ± 3.6d# | L |
| **Largest (Mbp)** |  |  |  |  |  |  |  |
| 150PE | 14 ± 0.5a | 25 ± 0.4b | 30 ± 2.6b | 31 ± 3.5b | 32 ± 5.3b | 29 ± 6.4b | L |
| 250PE | 26 ± 0.4# | 25 ± 4.2 | 27 ± 2.2 | 25 ± 0.8 | 29 ± 2.9 | 25 ± 0.8 |  |
| **N50** |  |  |  |  |  |  |  |
| 150PE | 373 ± 0a | 375 ± 3a | 402 ± 1b | 386 ± 0c | 386 ± 1c | 425 ± 1d | L |
| 250PE | 363.0 ± 2.6a# | 360 ± 2a# | 388 ± 2b# | 380 ± 1c# | 381 ± 1c# | 402 ± 4d# | L |
| **N90** |  |  |  |  |  |  |  |
| 150PE | 312 ± 0a | 313 ± 1a | 316 ± 0b | 314 ± 0c | 314 ± 0c | 317 ± 1d | L |
| 250PE | 310.3 ± 0.6a# | 310 ± 0a# | 318 ± 1b# | 314 ± 0c | 314 ± 0c | 329 ± 2d# | L |

**Supplementary Table 1-a.** **SRS** **Illumina assembly results per replicate.** Summatory bases of the assembly, number of contigs, average of contigs, largest contigs, N50 and N50 values of the different Illumina library preparation kits sequenced by 150PE or 250PE read length mode.

Parameters were calculated with the software assembly-stats (GitHub repository sanger-pathogens / assembly-stats). Results represent mean ± SEM (n = 3). Statistics: L, effect of the type of library preparation (one-way ANOVA, p < 0.05, and indicated when different). Values not sharing a common letter (a, b, c, d) are significantly different (one-way ANOVA, p < 0.05). Letter “a” is used to indicate the first values. No letters = no statistical difference. #, different vs. 150PE group (Student's t test, p < 0.05).

**Supplementary Table 1-b. SRS Illumina genes results per replicate.** Total number of genes, completed genes, percentage of completed genes, percentage of partial 3' genes, percentage of partial 5' genes and percentage of partial both sides genes of the different Illumina library preparation kits sequenced by 150PE or 250PE read length mode.

|  | **NEB_1** | **NEB_100** | **NEXT_1** | **Ovation_1** | **Ovation_100** | | **TruSeq_100** | **ANOVA** |
| --- | --- | --- | --- | --- | --- | --- | --- | --- |
| **Total number of genes (10^3)** |  |  |  |  |  |  | |  |
| 150PE | 274 ± 54a | 320 ± 35b | 380 ± 7c | 390 ± 6c | 402 ± 14c | 337 ± 9b | | L |
| 250PE | 1307 ± 11a# | 1413 ± 208a# | 4630 ± 132b# | 3057 ± 19c# | 3177 ± 142c# | 7208 ± 128d# | | L |
| **Completed genes (10^3)** |  |  |  |  |  |  | |  |
| 150PE | 3.26 ± 0.08a | 4.07 ± 0.49b | 7.91 ± 0.04c | 5.81 ± 0.16d | 6.05 ± 0.33d | 8.16 ± 0.15c | | L |
| 250PE | 14.1 ± 0.8a# | 15.3 ± 2.3a# | 71.9 ± 3.3b# | 38.7 ± 0.6c# | 41.1 ± 2.5c# | 120.9 ± 1.3d# | | L |
| **% of completed genes** |  |  |  |  |  |  | |  |
| 150PE | 1.18 ± 0.01a | 1.27 ± 0.01b | 2.08 ± 0.03c | 1.49 ± 0.02d | 1.50 ± 0.03d | 2.42 ± 0.03c | | L |
| 250PE | 1.1 ± 0.01a# | 1.1 ± 0.01a# | 1.55 ± 0.03b# | 1.27 ± 0.01c# | 1.29 ± 0.02c# | 1.68 ± 0.04d# | | L |
| **% of partial 3' genes** |  |  |  |  |  |  | |  |
| 150PE | 21.1 ± 0.01a | 21.4 ± 0.1b | 22.1 ± 0.01c | 21.5 ± 0.01d | 21.5 ± 0.01d | 22.6 ± 0.1e | | L |
| 250PE | 21.6 ± 0.10a# | 21.7 ± 0.02a# | 22.0 ± 0.1b# | 22.0 ± 0.03b# | 22.1 ± 0.05b# | 22.9 ± 0.04c# | | L |
| **% of partial 5' genes** |  |  |  |  |  |  | |  |
| 150PE | 21.8 ± 0.10a# | 21.9 ± 0.02a# | 22.3 ± 0.11b# | 22.3 ± 0.01b# | 22.4 ± 0.05b# | 23.2 ± 0.16c# | | L |
| 250PE | 21.1 ± 0.01a | 21.6 ± 0.01b | 22.4 ± 0.01c | 21.8 ± 0.06d | 21.7 ± 0.04d | 22.8 ± 0.08e | | L |
| **% of partial both side genes** |  |  |  |  |  |  | |  |
| 150PE | 56.5 ± 0.17 | 55.7 ± 0.05 | 53.4 ± 0.12 | 55.2 ± 0.01 | 55.2 ± 0.03 | 52.1 ± 0.16 | | L |
| 250PE | 55.6 ± 0.34a# | 55.2 ± 0.05a# | 54.2 ± 0.11b# | 54.5 ± 0.03b# | 54.2 ± 0.12b# | 52.1 ± 0.27c | | L |

Gene lists were obtained from Prodigal using the option “-p meta”. Results represent mean ± SEM (n = 3). Statistics: L, effect of the type of library preparation (one-way ANOVA, p < 0.05, and indicated when different). Values not sharing a common letter (a, b, c, d) are significantly different (one-way ANOVA, p < 0.05). Letter “a” is used to indicate the first values. #, different vs. 150PE group (Student's t test, p < 0.05).

**Supplementary Table 1-c. SRS co-assemblies and LRS assembly results**. Number of total reads used, summatory bases of the assembly, number of contigs, average of contigs, largest contigs, N50 and N90 values of the different SRS Illumina library preparation kits sequenced by 150PE or 250PE read length mode.

|  | **Number of reads**  **(in million)** | **Sum of bases**  **(in million)** | **Number of**  **contigs (10^3)** | **Average (bp)** | **Largest (Mbp)** | **N50** | **N90** | **HQ MAGs**  **(by MetaWrap)** |
| --- | --- | --- | --- | --- | --- | --- | --- | --- |
| **PacBio** | 1.7 | 531 | 15 | 35,894 | 8269 | 36,899 | 22,206 | NA |
| **All reads downsampling (GM)** |  |  |  |  |  |  |  |  |
| 150PE | 216 | 5,288 | 8,426 | 628 | 457 | 642 | 339 | NA |
| 250PE | 216 | 20,448 | 40,048 | 511 | 303 | 465 | 331 | NA |
| **Per kit (KM)** |  |  |  |  |  |  |  |  |
| **NEB-1** |  |  |  |  |  |  |  |  |
| 150PE | 38 | 518 | 1,188 | 436 | 26 | 405 | 316 | 4 |
| 250PE | 38 | 1,295 | 3,215 | 403 | 26 | 382 | 313 | 3 |
| **NEB-100** |  |  |  |  |  |  |  |  |
| 150PE | 38 | 532 | 1,226 | 434 | 25 | 403 | 315 | 3 |
| 250PE | 38 | 1,437 | 3,563 | 403 | 25 | 380 | 312 | 4 |
| **NEXT-1** |  |  |  |  |  |  |  |  |
| 150PE | 38 | 747 | 1,552 | 481 | 29 | 446 | 320 | 7 |
| 250PE | 38 | 4,794 | 1,1052 | 434 | 60 | 407 | 321 | 10 |
| **Ovation-1** |  |  |  |  |  |  |  |  |
| 150PE | 38 | 763 | 1,668 | 458 | 49 | 425 | 318 | 6 |
| 250PE | 38 | 3,237 | 7,683 | 421 | 49 | 398 | 317 | 10 |
| **Ovation-100** |  |  |  |  |  |  |  |  |
| 150PE | 38 | 787 | 1,717 | 458 | 41 | 426 | 318 | 7 |
| 250PE | 38 | 3,348 | 7,935 | 422 | 46 | 399 | 317 | 10 |
| **TruSeq-100** |  |  |  |  |  |  |  |  |
| 150PE | 38 | 731 | 1435 | 510 | 73 | 482 | 322 | 9 |
| 250PE | 38 | 7298 | 16253 | 449 | 92 | 418 | 332 | 12 |

Parameters were calculated with the software assembly-stats (GitHub repository sanger-pathogens / assembly-stats). Results represent unique value per kit and sequencing length.

|  | **MAG1** | | **MAG2** | | **MAG3** | | **MAG4** | |
| --- | --- | --- | --- | --- | --- | --- | --- | --- |
| Sequencing platform | Truseq_250 | PacBio | Truseq_250 | PacBio | Truseq_250 | PacBio | Truseq_250 | PacBio |
| % ANI | 100.0 | | 99.9 | | 99.8 | | 99.9 | |
| MASH Distance (p value) | 0.0036 | | 0.0033 | | 0.0048 | | 0.0059 | |
| MAG Quality | Medium | High | High | High | High | High | Medium | High |
| Completeness | 86.02 | 96.24 | 92.67 | 96.26 | 95.33 | 90.74 | 85.14 | 93.53 |
| Contamination | 3.42 | 3.23 | 3.88 | 0.86 | 0.00 | 0.00 | 1.14 | 0.00 |
| N50 (Mb) | 12 | 8,268 | 12 | 605 | 24 | 371 | 8.7 | 276 |
| rRNA (16S, 23S, 5S) | 1, 0, 0 | 1, 1, 1 | 1, 0, 1 | 1, 1, 1 | 1, 1, 1 | 1, 1, 1 | 1, 0, 1 | 1, 1, 1 |
| tRNA | 57 | 71 | 44 | 47 | 51 | 45 | 28 | 40 |
| Total Length (Kb) | 7.2 | 8.3 | 2.0 | 2.7 | 8.4 | 8.3 | 2.2 | 2.6 |
| L50 | 180 | 1 | 65 | 2 | 104 | 8 | 86 | 4 |
| N contigs | 833 | 1 | 279 | 7 | 542 | 36 | 310 | 13 |
| N predicted genes (prokka) | 5,706 | 6,136 | 2,592 | 2,688 | 6,812 | 6,818 | 2,102 | 2,571 |
| Mean contig coverage | 9.3 | 8.0 | 6.6 | 4.4 | 7.9 | 3.4 | 5.4 | 3.2 |

**Supplementary Table 1-d. SRS KM-TruSeq-250PE and LRS PacBio MAG results comparison**. % of ANI, p value of the MASH distance, MAG quality, % of completeness and contamination, rRNA and tRNA gene content, N50 (Mb), total of the genome assembled (Kb), L50, number of contigs, number of predicted genes and mean contig coverage of the SRS KM-Truseq-250PE and LRS PacBio.
