# Supplementary material for: Comparative metagenomic assessment of Illumina-compatible library preparation methods, short-read lengths, and PacBio HiFi sequencing reveals differences in microbial and functional diversity recovery from a complex environmental sample
Source: Microbiol Spectr. 2026 Jul 27;14(9):e00013-26. doi: 10.1128/spectrum.00013-26 (PMC13532183; doi:10.1128/spectrum.00013-26)
Supplement: Supplementary file 6 [file spectrum.00013-26-s0008.docx]

|  | | **NEB_100** | **NEB_100-filtered** | **NEXT_1** | **TruSeq_100** | **ANOVA** |
| --- | --- | --- | --- | --- | --- | --- |
| **Sum of assembly (Mbp)** | |  |  |  |  |  |
| 250PE | | 472 ± 71a | 1084 ± 180b | 1637 ± 52c | 2605 ± 34d | L |
| **Contigs (10^6)** | |  |  |  |  |  |
| 250PE | | 12.5 ± 1.8a | 28.4 ± 4.5b | 40.7 ± 1.1c | 62.6 ± 1.3d | L |
| **Average (bp)** | |  |  |  |  |  |
| 250PE | | 376 ± 1.5a | 387± 3.0b | 402 ± 1.7c | 416 ± 3.6d | L |
| **Largest (Mbp)** | |  |  |  |  |  |
| 250PE | | 25 ± 4.2 | 25 ± 2.5 | 27 ± 2.2 | 25 ± 0.8 |  |
| **N50** | |  |  |  |  |  |
| 250PE | | 360 ± 2a | 369 ± 2b | 388 ± 2c | 402 ± 4d | L |
| **N90** | |  |  |  |  |  |
| 250PE | | 310 ± 0a | 311 ± 0a | 318 ± 1b | 329 ± 2c | L |
| **Total number of genes (10^3)** | |  |  |  |  |  |
| 250PE | | 1413 ± 208a | 3178 ± 513b | 4630 ± 132c | 7208 ± 128d | L |
| **% of completed genes** | |  |  |  |  |  |
| 250PE | | 1.1 ± 0.01a | 1.3 ± 0.1b | 1.55 ± 0.03c | 1.68 ± 0.04d |  |
|  | |  |  |  |  |  |
|  | |  |  |  |  |  |
|  |  |  |  |  |  |  |

**Supplementary Table 4.**

A) **Illumina assembly and gene results.** Summatory bases of the assembly, number of contigs, average of contigs, largest contigs, N50 and L50 values of the different Illumina library preparation kits sequenced by 250PE read length mode.

Parameters were calculated with the software assembly-stats (GitHub repository sanger-pathogens / assembly-stats). Results represent mean ± SEM (n = 3). Statistics: L, effect of the type of library preparation (one-way ANOVA, p < 0.05, and indicated when different). Values not sharing a common letter (a, b, c, d) are significantly different (one-way ANOVA, p < 0.05). Letter “a” is used to indicate the first values. No letters = no statistical difference.

B) **Illumina assembly, genes and MAGs results for the kit co-assemblies.** Summatory bases of the assembly, number of contigs, average of contigs, largest contigs, N50, total number of detected genes, total recovered MAGs and High-quality MAGs values of the different Illumina library preparation kits sequenced by 2 x 250 bp read length mode.

|  | **NEB_100** | **NEB_100-filtered** | **NEXT_1** | **Truseq_100** |
| --- | --- | --- | --- | --- |
| **Sum of assembly (in million)** |  |  |  |  |
| 2 x 250 bp | 1,437 | 3,409 | 4,794 | 7,298 |
| **Contigs (10^3)** |  |  |  |  |
| 2 x 250 bp | 3,563 | 8,111 | 11,052 | 16,253 |
| **Average (bp)** |  |  |  |  |
| 2 x 250 bp | 403 | 420 | 434 | 449 |
| **Largest (Mbp)** |  |  |  |  |
| 2 x 250 bp | 25 | 45 | 60 | 92 |
| **N50** |  |  |  |  |
| 2 x 250 bp | 380 | 393 | 407 | 418 |
| **Total detected genes (in million)** |  |  |  |  |
| 2 x 250 bp | 4.1 | 9.4 | 12.8 | 19,2 |
| **Total MAGs** |  |  |  |  |
| 2 x 250 bp | 6 | 13 | 29 | 36 |
| **High quality MAGs** |  |  |  |  |
| 2 x 250 bp | 1 | 3 | 6 | 11 |

Parameters were calculated with the software assembly-stats (GitHub repository sanger-pathogens / assembly-stats). Results represent unique value per kit.
